# Lycoplanines B-D, Three *Lycopodium* Alkaloids from *Lycopodium complanatum*

**DOI:** 10.1007/s13659-018-0161-2

**Published:** 2018-04-09

**Authors:** Zhi-Jun Zhang, Qin-Feng Zhu, Jia Su, Xing-De Wu, Qin-Shi Zhao

**Affiliations:** 1 State Key Laboratory of Phytochemistry and Plant Resources in West China, Kunming Institute of Botany, Chinese Academy of Sciences, Kunming, 650204 People’s Republic of China; 2 University of Chinese Academy of Sciences, Beijing, 100049 People’s Republic of China

**Keywords:** *Lycopodium complanatum*, Lycoplanines, *Lycopodium alkaloids*, Anti-AChE activity

## Abstract

**Abstract:**

A novel C_17_N *Lycopodium* alkaloid (LA), lycoplanine B (**1**), containing an unusual formyl group, along with two new LAs, lycoplanines C (**2**) and D (**3**), were isolated from the whole plant of *Lycopodium complanatum*. Their structures were elucidated by extensive NMR techniques, including 1D- and 2D-NMR experiments, as well as comparing their spectral data with those of the known analogues. A possible biogenetic pathway for **1** was also proposed.

**Graphical Abstract:**

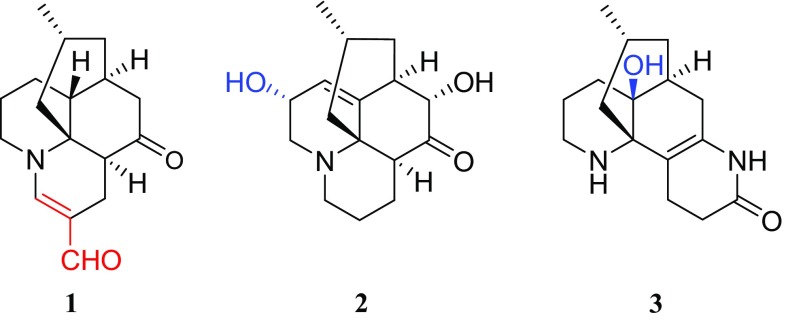

**Electronic supplementary material:**

The online version of this article (10.1007/s13659-018-0161-2) contains supplementary material, which is available to authorized users.

## Introduction

*Lycopodium complanatum* belonging to the family Lycopodiaceae, is mainly distributed in temperate and subtropical areas. This plant has been used as a folk medicine for the treatment of arthritic pain, quadriplegia, contusion, and blood stasis [1, 2]. Previous phytochemical investigations of this plant led to the isolation of a number of *Lycopodium* alkaloids (LAs) [3–6], which have attracted great interest from biogenetic [7, 8], synthetic [9–11], and biological [12, 13] points of view.

**Fig. 1:**
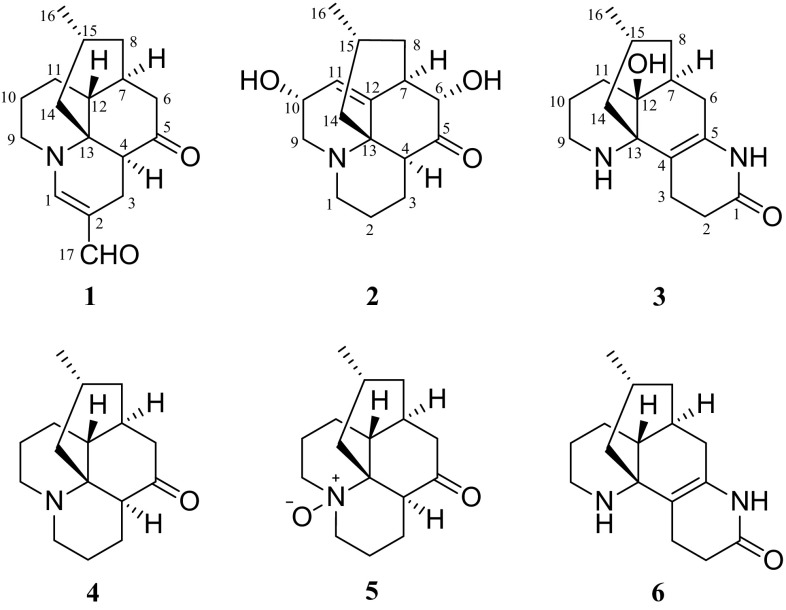
Structures of compounds **1**–**5**

In our continuing efforts to find biogenetically interesting and structurally unique LAs, we reported a LA, lycoplanine A, posessing an intriguing tricyclic (6/9/5) ring skeleton from *L. complanatum* [14]. Further chemical investigation of the extracts of this plant resulted in the isolation of three new LAs, lycoplanines B-D (**1**–**3**), along with three known compounds lycopodine (**4**), lycopodine *N*-oxide (**5**), and des-*N*-methyl-*α*-obscurine (**6**). In current study, we describe the isolation, structure elucidation, and anti-AChE activity of **1**–**3** (Fig. 1), as well as a plausible biogenetic path for lycoplanine B (**1**).

## Results and Discussion

The air-dried and powdered whole plant of *L. complanatum* was extracted with EtOH three times. The extract was partitioned between EtOAc and 1.0‰ HCl/H_2_O. The pH of the water-soluble portion was adjusted to pH 9 with saturated Na_2_CO_3_ aq. Then, it was extracted with CHCl_3_ to afford an alkaloidal extract. Further column chromatography over MCI gel, silica gel, Sephadex LH-20, and RP-18 led to the isolation of compounds **1** (5 mg), **2** (3 mg), **3** (6 mg), **4** (30 mg), and **5** (8 mg), and **6** (15 mg).

Lycoplanine B (**1**), $[\alpha]_{22.3}^{D}$ −166.85 (*c* 0.18, MeOH), white amorphous powder, gave rise to a quasi-molecular ion peak at *m/z* 274.1806 ([M+H]^+^) in the HRESIMS, which corresponded to the molecular formula C_17_H_23_NO_2_, and was further confirmed by its ^13^C NMR and DEPT analysis. The IR absorption bands indicated the existence of carbonyl (1703 cm^−1^), and olefinic functional (1637 cm^−1^) groups. The ^13^C NMR spectroscopic data of **1** (Table 1), with the aid of an HSQC NMR experiment, showed 17 carbons due to one methyl (*δ*_C_ 22.8), seven *sp*^3^ methylenes, one *sp*^2^ (*δ*_C_ 158.6) methine, four *sp*^3^ methines, one *sp*^3^ (*δ*_C_ 62.2) quaternary carbon, one *sp*^2^ (*δ*_C_ 113.2) quaternary carbon, one carbonyl group (*δ*_C_ 212.1), and one aldehyde group (*δ*_C_ 189.4), of which one *sp*^3^ quaternary carbon (*δ*_C_ 62.2) and one *sp*^3^ methylene carbon (*δ*_C_ 49.1) were attributed to those attached to a nitrogen atom.

**Table 1:** ^1^H and ^13^C NMR spectroscopic data of compounds **1**–**3** (*δ* in ppm)

No.	**1** ^a^		**2** ^b^		**3** ^a^	
	*δ*_H_ (*J* in Hz)	*δ*_C_	*δ*_H_ (*J* in Hz)	*δ*_C_	*δ*_H_ (*J* in Hz)	*δ*_C_
1a	7.24 (s)	158.6 d	2.95 (m)	47.3 t		173.7 s
1b			2.53 (d, 11.0)			
2a		113.2 s	1.69 (m)	24.0 t	2.47 (m)	31.7 t
2b			1.69 (m)		2.43 (m)	
3a	2.01 (m)	20.1 t	2.00 (d, 13.9)	19.2 t	2.36 (m)	20.4 t
3b	1.65 (m)		1.56 (m)		2.32 (m)	
4	2.54 (dd, 11.9, 3.2)	51.7 d	2.95 (m)	50.9 d		113.5 s
5		212.1 s		210.1 s		133.4 s
6a	2.78 (dd, 16.5, 6.4)	43.1 t	3.87 (d, 2.0)	77.9 d	2.50 (m)	33.3 t
6b	2.16 (d, 16.5)				1.95 (m)	
7	2.36 (m)	37.4 d	2.72 (d, 2.0)	47.6 d	1.85 (m)	40.3 d
8a	1.76 (m)	43.3 t	1.85 (d, 11.6)	39.6 t	1.46 (m)	39.8 t
8b	1.33 (m)		1.28 (td, 11.6, 4.9)		1.23 (m)	
9a	3.67 (td, 13.4, 3.2)	49.1 t	2.92 (d, 12.1)	53.4 t	2.81 (m)	43.1 t
9b	3.27 (m)		2.65 (dd, 12.1, 3.0)		2.56 (td, 12.7, 3.1)	
10a	1.81 (m)	27.8 t	4.04 (d, 3.0)	64.3 d	1.99 (m)	23.0 t
10b	1.48 (m)				1.46 (m)	
11a	2.43 (m)	20.5 t	5.82 (d, 4.5)	123.2 d	1.99 (m)	32.5 t
11b	2.36 (m)				1.42 (m)	
12	1.88 (m)	41.7 d		143.5 s		70.3 s
13		62.2 s		60.2 s		60.5 s
14a	2.67 (td, 13.2, 4.0)	41.7 t	2.20 (dd, 13.0, 3.2)	33.1 t	1.83 (m)	38.5 t
14b	0.93 (m)		1.09 (t, 13.0)		1.36 (m)	
15	1.48 (m)	26.8 d	1.56 (m)	25.4 d	1.71 (m)	27.2 d
16	0.88 (d, 6.2)	22.8 q	0.84 (d, 6.1)	22.5 q	0.92 (d, 6.5)	22.3 q
17	8.73 (s)	189.4 d				

Assignments were based on ^1^H–^1^H COSY, HSQC, and HMBC experiments. ^1^H and ^13^C NMR were measured at 600 and 150 MHz, respectively

^a^Samples were in CD_3_OD

^b^Samples were in CDCl_3_

**Fig. 2:**
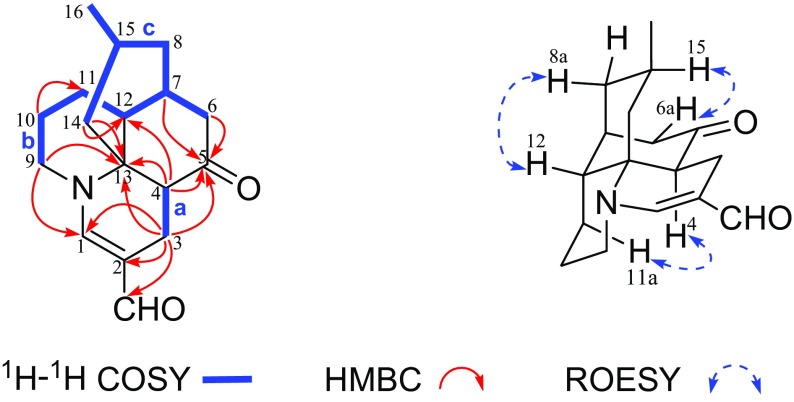
Key 2D-NMR correlations for compound **1**

The gross structure of **1** was elucidated by analyses of 2D NMR data including ^1^H–^1^H COSY, HSQC, and HMBC spectra recorded in CD_3_OD (Fig. 2). The ^1^H-^1^H COSY spectrum of **1** revealed the presence of two fragments: **a** (C-3/C-4) and **b** (C-9/C-10/C-11/C-12/C-7/C-6, C-7/C-8/C-15/C-16 and C-15/C-14). The connection of C-4 and C-6 through the ketone group (C-5) as evidenced by the HMBC correlations from H-3, H-4, H-6, and H-7 to a ketone group (*δ*_C_ 212.1). The connection of C-4 and C-12 through quaternary carbon (C-13) as evidenced by the HMBC correlations of H-3 and H-4 with quaternary carbon (*δ*_C_ 62.2), and H-4 with C-12. What’s more, C-3 and C-17 was connected by *sp*^2^ quaternary carbon (C-2), which was also elucidated by the HMBC correlations from H-3 to C-1, C-2, and C-17. Meanwhile, HMBC cross-peaks of H-9 with C-1 and C-13 established the connections of C-1, C-9, and C-13 through a nitrogen atom. In addation, HMBC correlations from H-10 to C-11 and C-12, H-14 to C-4, C-12, C-13, C-15, and C-16 could be observed. Thus, the basic planar structure of **1** was determined.

The relative configuration of **1** was elucidated by the ROESY spectrum (Fig. 2) and biogenetic consideration of lycopodine-type alkaloid derivatives isolated from Lycopodiaceae family. The cross-peaks of H-12 with H-8a, H-15 with H-6a were observed in ROESY experiment indicating the *β*-orientation of H-12 and H-15. The correlation of H-4 with H-11b indicated the *α*-orientation of H-4. Thus, the structure of compound **1** was characterized as 2-formyl-1,2-dehydro-lycopodine.

**Fig. 3:**
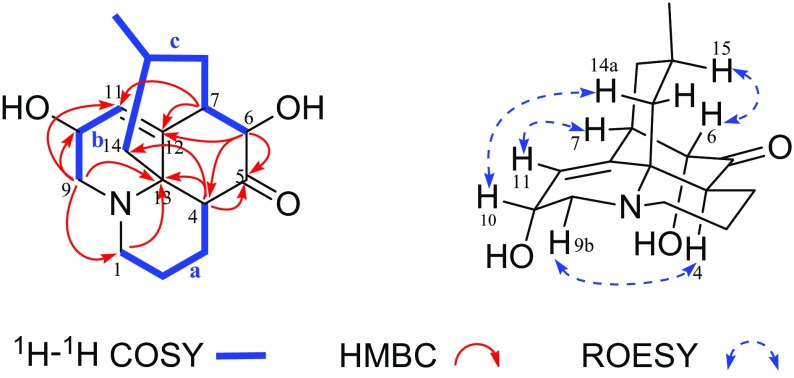
Key 2D-NMR correlations for compound **2**

Lycoplanine C (**2**), obtained as colorless oil, had a molecular formula of C_16_H_23_NO_3_ by the HRESIMS ion peak at *m/z* 278.1758 [M+H]^+^ (calcd for C_16_H_24_NO_3_, 278.1751) with six degrees of unsaturation. The IR absorption at 3414 cm^−1^ suggested the presence of hydroxy group. In agreement with its molecular formula, all the sixteen carbon signals were observed in the ^13^C NMR spectrum (Table 1), and were further classified by DEPT experiments into one methyl (*δ*_C_ 22.5), six methylenes, six methines (one olefinic at *δ*_C_ 123.2), and three quaternary carbons (one olefinic at *δ*_C_ 143.5, and one carbonyl at *δ*_C_ 210.1). The ^1^H NMR (Table 1) spectrum of **2** displayed one methyl (*δ*_H_ 0.84, d, *J* = 6.1 Hz) and one olefinic proton (*δ*_H_ 5.82, d, *J* = 4.5 Hz). Careful comparison of the NMR spectroscopic data of **2** with those of lycoposerramine K [15] suggested that they possessed the same carbon skeleton. The fragments of **a** C-1/C-2/C-3/C-4, **b** C-9/C-10/C-11, and **c** C-6/C-7/C-8/C-15(C-16)/C-14 (Fig. 3) determined by the ^1^H-^1^H COSY correlations (Fig. 3), in combination with a series of HMBC correlations (Fig. 3) of H-9 with C-1 and C-13, H-1 with C-9 and C-13, H-4 with C-5, C-12 and C-13, H-6 with C-4, C-5 and C-12, and H-7 with C-11 and C-12, further confirmed the above deduction. The only difference between **2** and lycoposerramine K was that **2** possessed an additional hydroxy group connected to C-10, as inferred from the HMBC correlations of H-9 and H-11 with C-10 (*δ*_C_ 64.3).

The relative configuration of **2** was assigned on the basis of ROESY experiment. The ROESY correlations (Fig. 3) of H-15 with H-6, H-11 with H-7, H-10 with H-14a, and H-4 with H-9b indicated that H-4 and H-7 were *α*-oriented, while H-6, H-10 and H-15 were *β*-oriented. Therefore, compound **2** was unambiguously established as 10*α*-hydroxy-lycoposerramine K.

Lycoplanine D (**3**) has the molecular formula C_16_H_24_N_2_O_2_ based on its HRESIMS (m/z 277.1914) ([M+H]^+^, calcd 277.1911) and ^13^C NMR spectroscopic data. The IR absorption bands indicated the existence of hydroxy (3495 cm^−1^), carbonyl (1698 cm^−1^), and olefinic functional (1657 cm^−1^) groups. The ^13^C NMR (Table 1) spectroscopic data of **3** showed the exitence of 16 signals classified as one methyl resonated at *δ*_C_ 22.3, eight methylenes, two methines, and five quaternary carbons (one amide, two olefinic carbons, and two *sp3* quaternary carbons). Further 2D NMR analysis indicated that **3** should be a lycodine-type alkaloid with many similarities to that of des-*N*-methyl-*α*-obscurine [16], which was also isolated in present study. Differing from des-*N*-methyl-*α*-obscurine, the molecular formula of **3** contains one more oxygen atom. Meanwhile, the methine group (*δ*_C_ 44.5) at C-12 in des-*N*-methyl-*α*-obscurine was replaced by a *sp3* quaternary carbon (*δ*_C_ 70.3) in **3**. So, the H-12 in des-*N*-methyl-*α*-obscurine was replaced by a hydroxyl group in **3**. The HMBC correlations of H-7, H-8, H-11, and H-14 with C-12 (*δ*_C_ 70.3) and C-13 (*δ*_C_ 60.5), further confirmed the above deduction. The relative configurations of 12-OH and H-15 were deduced to be *β*-oriented, according to the cross-peaks of H-11b/H-6b and H-15/H-6a in the ROESY spectrum. Therefore, compound **3** was determined as 12-hydroxy-des-*N*-methyl-*α*-obscurine.

**Scheme 1:**
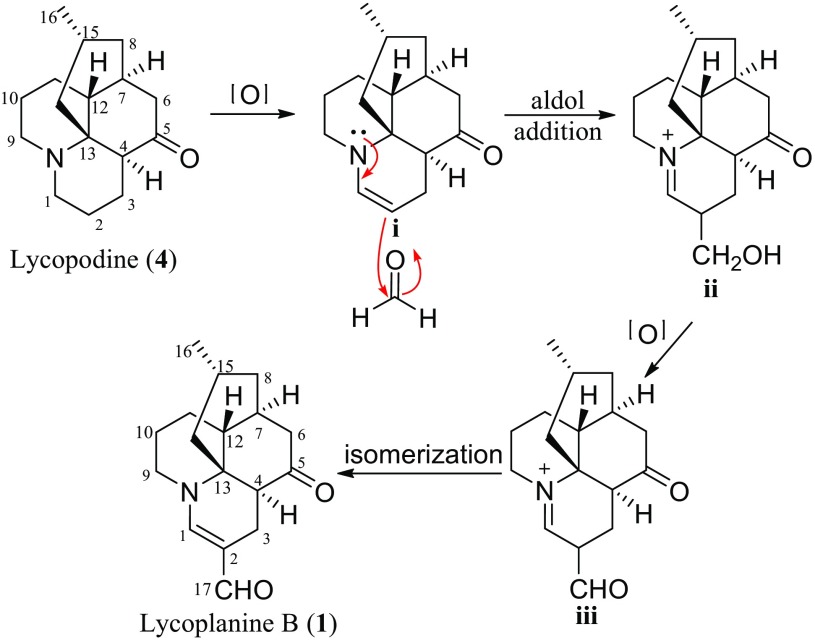
Plausible biogenetic formation of lycoplanine B (**1**)

Lycoplanine B (**1**), a novel C_17_N *Lycopodium* alkaloid, is the first example of *Lycopodium* alkaloids containing an unusual formyl group. A possible biogenetic pathway for **1** was also proposed as shown in Scheme 1. Compound **1** might be generated from lycopodine (**4**) [17], which was also isolated from the plant in current study. In brief, **4** underwent oxidation step to produce intermediate **i**. Intermediate **i** give **ii** through a aldol addition reaction. Intermediate **ii** could then underwent oxidation reaction to yield key intermediate **iii**. Then, intermediate **iii** accompanied by a isomerization behaviors of the double bond to get **1**.

Compounds **1**–**3** were also tested for AChE inhibitory activities using the Ellman method [18] reported previously (huperzine A as positive control, IC_50_ = 0.03 μM). Unfortunately, they showed no inhibitory activity (IC_50_ > 100 μM) after two repeated experiments.

## Experimental

### General

Optical rotations were measured with a Horiba SEPA-300 polarimeter (Horiba, Tokyo, Japan). UV spectra were obtained using a Shimadzu UV-2401A spectrophotometer (Shimadzu, Tokyo, Japan). IR spectra were obtained by a Tenor 27 spectrophotometer (Bruker Optics, Ettlingen, Germany) using KBr pellets. 1D and 2D NMR spectra were carried out on Bruker AM-400, DRX-500, or AVANCE III-600 spectrometers with TMS as an internal standard (Bruker, Karlsruhe, Germany). ESIMS and HRESIMS were run on an Agilent 6530 Q of spectrometer (Agilent, Palo Alto, CA, USA). Column chromatography (CC) was performed using MCI gel (CHP 20P, 75–150 m; Mitsubishi Chemical Corporation, Tokyo, Japan), Polyamide (PA, 80–100 mesh; Sinopharm Chemical Reagent Co., Ltd, Shanghai, China) and silica gel (100–200 or 200–300 mesh; Qingdao Haiyang Chemical Co., Ltd, Qingdao, China). Thin-layer chromatography (TLC) was conducted on silica gel plates GF_254_ (Qingdao Haiyang Chemical Co., Ltd, Qingdao, China). Fractions were monitored by TLC using various solvent systems, and spots were visualized by spraying improved Dragendorff’s reagent to the silica gel plates or by heating silica gel plates sprayed with 10% H_2_SO_4_ in EtOH.

### Plant Material

The whole plants of *Lycopodium complanatum* were collected in Guizhou Province, People’s Republic of China, in September 2013. The sample was identified by Dr. Xiao Cheng. A voucher specimen (20130915) has been deposited in the State Key Laboratory of Phytochemistry and Plant Resources in West China, Kunming Institute of Botany, Chinese Academy of Sciences, People’s Republic of China.

### Extraction and Isolation

The air-dried and powdered sample (30 kg) was extracted with 60% EtOH (24 h × 3), the extract was partitioned between EtOAc and 1.0‰ HCl/H_2_O. Water-soluble materials, which were adjusted to pH 10 with saturated Na_2_CO_3_ aq, were extracted with CHCl_3_ to give an alkaloidal extract (60 g). The alkaloidal extract was subjected to medium pressure liquid chromatography (MPLC) over RP-18 gel and eluted with MeOH/H_2_O (1:4–1:0) to yield five subfractions A-E. Fraction B (10 g) was chromatographed over silica gel CC (petroleumether/Me_2_CO, 8:1–1:1) to afford five subfractions, fractions B1-B6. Fraction B3 (3.5 g) was purified by silica gel CC (CHCl_3_/MeOH, 80:1–10:1) to obtain **1** (5 mg). Fraction C (18 g) was subjected to silica gel CC (petroleum ether/Me_2_CO, from 9:1 to 4:1) to give six subfractions, fractions C1-C6. Fraction C3 (5.4 g) was submitted to silica gel CC (CHCl_3_/MeOH, 50:1–10:1) to yield **2** (3 mg) and **4** (30 mg). Fraction D (11 g) was successively subjected to RP-18 (30% MeOH/H_2_O) to give three subfractions, fractions D1-D3. Fraction D3 (4.5 g) was further purified by a silica gel column chromatography (CHCl_3_/MeOH, 200:1) and Sephadex LH-20 to afford **3** (6 mg) and **5** (8 mg).

#### Lycoplanine B (**1**)

White amorphous powder; $[\alpha]_{22.3}^{D}$ −166.85 (*c* 0.18, MeOH); IR (KBr) υ_max_ 1703, 1637, 1600, 1412 cm^−1^; UV (MeOH) λ_max_ (log ε) 202 (3.80), 302 (4.36) nm; ^1^H- and ^13^C-NMR spectroscopic data, see Table 1; HRESIMS (pos.) *m/z* 274.1806 ([M+H]^+^) (calcd for C_17_H_24_NO_2_, 274.1802).

#### Lycoplanine C (**2**)

Colorless oil; $[\alpha]_{22.3}^{D}$ −50.28 (*c* 0.12, MeOH); IR (KBr) υ_max_ 3414, 2925, 1711, 1454, 1043, cm^−1^; UV (MeOH) λ_max_ (log ε) 203 (3.68) nm; ^1^H- and ^13^C-NMR spectroscopic data, see Table 1; HRESIMS (pos.) *m/z* 278.1758 ([M+H]^+^) (calcd for C_16_H_24_NO_3_, 278.1751).

#### Lycoplanine D (**3**)

White amorphous powder; $[\alpha]_{22.3}^{D}$ −26.58 (*c* 0.08, MeOH); IR (KBr) υ_max_ 3495, 1657, 1384, 1196 cm^−1^; UV (MeOH) λ_max_ (log ε) 201 (3.62), 253 (3.78) nm; ^1^H- and ^13^C-NMR spectroscopic data, see Table 1; HRESIMS (pos.) *m/z* 277.1914 ([M+H]^+^) (calcd for C_16_H_25_N_2_O_2_, 277.1911).

### Acetylcholinesterase Inhibition Activity

Acetylcholinesterase (AChE) inhibitory activity of compounds **1**–**3** were assayed by the spectrophotometric method developed by Ellman et al. with slightly modification (Ellman et al. 1961). *S*-Acetylthiocholine iodide, *S*-butyrylthiocholine iodide,5,5′-dithio-bis-(2-nitrobenzoic) acid (DTNB, Ellman’s reagent), acetylcholinesterase derived from human erythrocytes were purchased from Sigma Chemical. Compounds **1**–**3** were dissolved in DMSO. The reaction mixture (totally 200 μL) containing phosphate buffer (pH 8.0), test compound (50 μM), and acetyl cholinesterase (0.02 U/mL), was incubated for 20 min (30 °C). Then, the reaction was initiated by the addition of 40 μL of solution containing DTNB (0.625 mM) and acetylthiocholine iodide (0.625 mM) for AChE inhibitory activity assay, respectively. The hydrolysis of acetylthiocholine was monitored at 405 nm every 30 s for 1 h. Tacrine was used as positive control with final concentration of 0.333 μM. All the reactions were performed in triplicate. The percentage inhibition was calculated as follows:  % inhibition = (E − S)/E × 100 (E is the activity of the enzyme without test compound and S is the activity of enzyme with test compound).

## Electronic supplementary material

Below is the link to the electronic supplementary material.

Supplementary material 1 (DOC 4052 kb)

## References

[1] Zhang XC, Zhang LB (2004). Flora of China. Beijing: Science Press.

[2] College Jiangsu New Medical (1986). Dictionary of the Tranditional Chinese Medicine.

[3] Kobayashi J, Hirasawa Y, Yoshida N, Morita H (2001). J. Org. Chem..

[4] Wu XD, He J, Xu G, Peng LY, Song LD, Zhao QS (2009). Acta Bot Yunnan.

[5] Ishiuchi KI, Kubota T, Ishiyama H, Hayashi S, Shibata T, Kobayashi J (2011). Tetrahedron Lett..

[6] Cheng JT, Liu F, Li XN, Wu XD, Dong LB, Peng LY, Huang SX, He J, Zhao QS (2013). Org. Lett..

[7] Ishiuchi KI, Kubota T, Mikami Y, Obara Y, Nakahata N, Kobayashi J (2007). Bioorg. Med. Chem..

[8] Ishiuchi KI, Kubota T, Ishiyama H, Hayashi S, Shibata T, Mori K, Obara Y, Nakahata N, Kobayashi J (2011). Bioorg. Med. Chem..

[9] Fischer DF, Sarpong R (2010). J. Am. Chem. Soc..

[10] Nakamura Y, Burke AM, Kotani S, Ziller JW, Rychnovsky SD (2010). Org. Lett..

[11] Cheng X, Waters SP (2010). Org. Lett..

[12] Liu JS, Zhu YL, Yu CM, Zhou YZ, Han YY, Wu FW, Qi BF (1986). Can. J. Chem..

[13] Vallejo MG, Ortega MG, Cabrera JL, Carlini VP, de Barioglio SR, Agnese AM (2007). J. Ethnopharmacol..

[14] Zhang ZJ, Nian Y, Zhu QF, Li XN, Su J, Wu J, Yang J, Zhao QS (2017). Org. Lett..

[15] Takayama H, Katakawa K, Kitajima M, Yamaguchi K, Aimi N (2003). Chem. Pharm. Bull..

[16] Ayer WA, Kasitu GC (1989). Can. J. Chem..

[17] Nakashima TT, Singer PP, Browne LM, Ayer WA (1975). Can. J. Chem..

[18] Ellman GL, Courtney KD, Andres V, Featherstone RM (1961). Biochem. Pharmacol..

