# Supplementary material for: Lycoplanines B-D, Three Lycopodium Alkaloids from Lycopodium complanatum
Source: Nat Prod Bioprospect. 2018 Apr 9;8(3):177–82. doi: 10.1007/s13659-018-0161-2 (PMC5971032; doi:10.1007/s13659-018-0161-2)
Supplement: Supplementary file 1 — Supplementary material 1 (DOC 4052 kb) [file 13659_2018_161_MOESM1_ESM.doc]

**Supplementary data to**

**Lycoplanines B-D, three *Lycopodium* alkaloids from *Lycopodium complanatum***

Zhi-Jun Zhang,^†,‡^ Qin-Feng Zhu,^†,‡^ Jia Su,^†^ Xing-De Wu,^†^ and Qin-Shi Zhao[[1]](#footnote-2)*,†

^†^ State Key Laboratory of Phytochemistry and Plant Resources in West China, Kunming Institute of Botany, Chinese Academy of Sciences, Kunming 650204, China

^‡^ University of Chinese Academy of Sciences, Beijing 100049, People’s Republic of China

**Table of Contents**

S1. HRESIMS spectrum of compound **1**

S2. ^1^H-NMR spectrum (600 MHz, methanol-*d_4_*) of compound **1**

S3. ^13^C-NMR spectrum (150 MHz, methanol-*d_4_*) of compound **1**

S4. ^1^H-^1^H COSY NMR spectrum (methanol-*d_4_*) of compound **1**

S5. ^1^H-^13^C HSQC NMR spectrum (methanol-*d_4_*) of compound **1**

S6. ^1^H-^13^C HMBC NMR spectrum (methanol-*d_4_*) of compound **1**

S7. ROESY spectrum (methanol-*d_4_*) of compound **1**

S8. UV spectrum of compound **1**

S9. OR spectrum of compound **1**

S10. IR spectrum of compound **1**

S11. HRESIMS spectrum of compound **2**

S12. ^1^H-NMR spectrum (600 MHz, methanol-*d_4_*) of compound **2**

S13. ^13^C-NMR spectrum (150 MHz, methanol-*d_4_*) of compound **2**

S14. ^1^H-^1^H COSY NMR spectrum (methanol-*d_4_*) of compound **2**

S15. ^1^H-^13^C HSQC NMR spectrum (methanol-*d_4_*) of compound **2**

S16. ^1^H-^13^C HMBC NMR spectrum (methanol-*d_4_*) of compound **2**

S17. ROESY spectrum (methanol-*d_4_*) of compound **2**

S18. UV spectrum of compound **2**

S19. OR spectrum of compound **2**

S20. IR spectrum of compound **2**

S21. HRESIMS spectrum of compound **3**

S22. ^1^H-NMR spectrum (600 MHz, methanol-*d_4_*) of compound **3**

S23. ^13^C-NMR spectrum (150 MHz, methanol-*d_4_*) of compound **3**

S24. ^1^H-^1^H COSY NMR spectrum (methanol-*d_4_*) of compound **3**

S25. ^1^H-^13^C HSQC NMR spectrum (methanol-*d_4_*) of compound **3**

S26. ^1^H-^13^C HMBC NMR spectrum (methanol-*d_4_*) of compound **3**

S27. ROESY spectrum (methanol-*d_4_*) of compound **3**

S28. UV spectrum of compound **3**

S29. OR spectrum of compound **3**

S30. IR spectrum of compound **3**


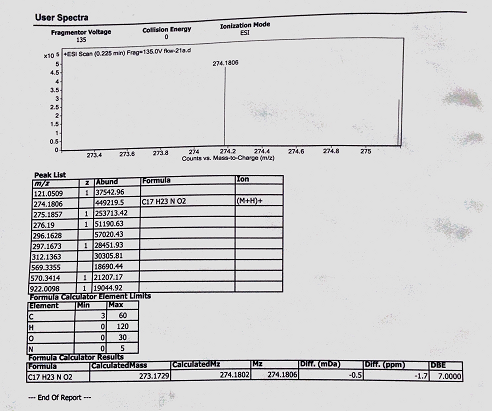


S1. HRESIMS spectrum of compound **1**


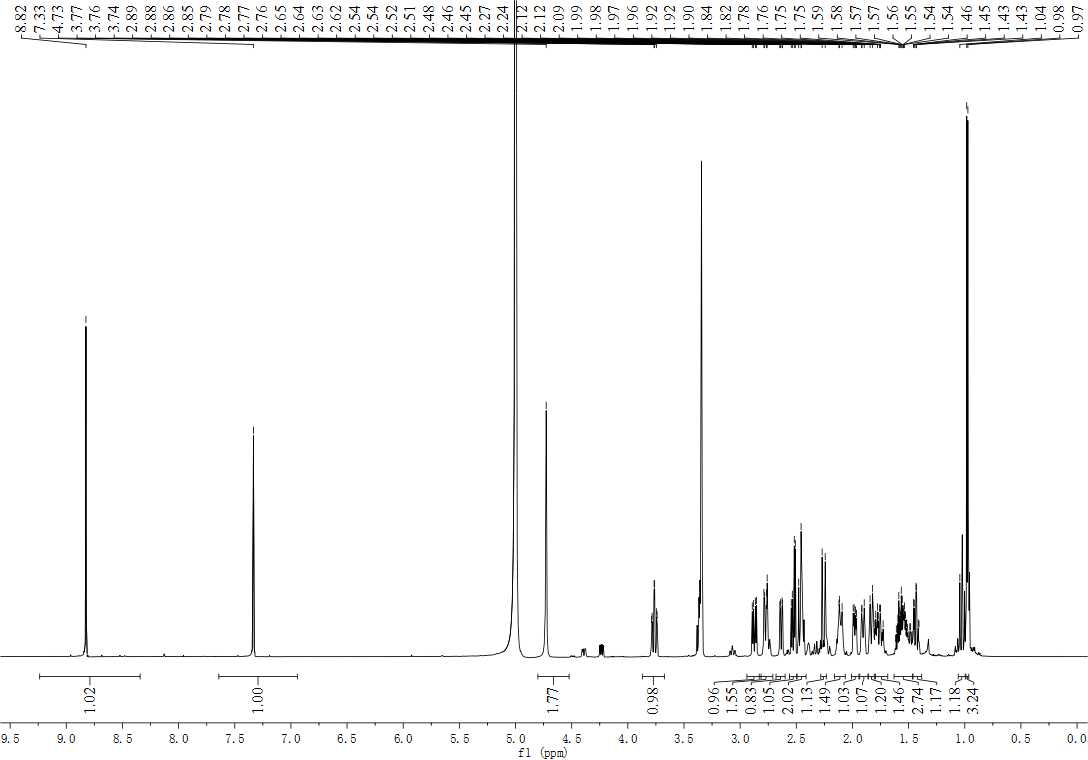


S2. ^1^H-NMR spectrum (600 MHz, methanol-*d_4_*) of compound **1**


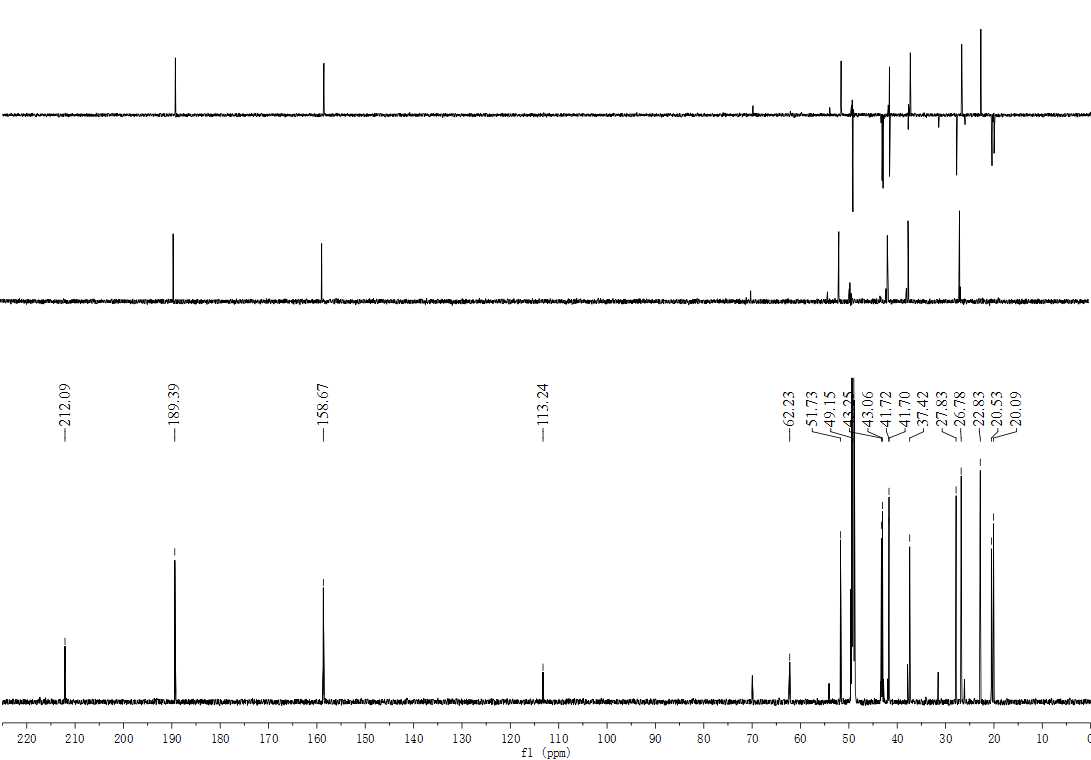


S3. ^13^C-NMR spectrum (150 MHz, methanol-*d_4_*) of compound **1**


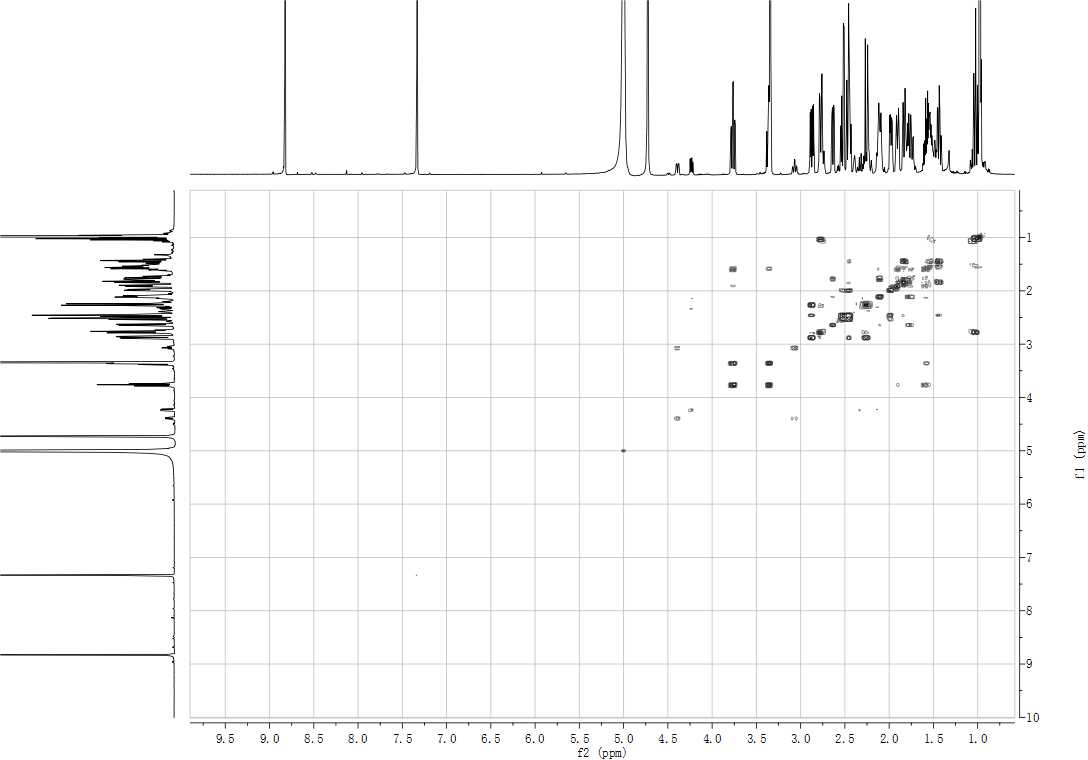


S4. ^1^H-^1^H COSY NMR spectrum (methanol-*d_4_*) of compound **1**


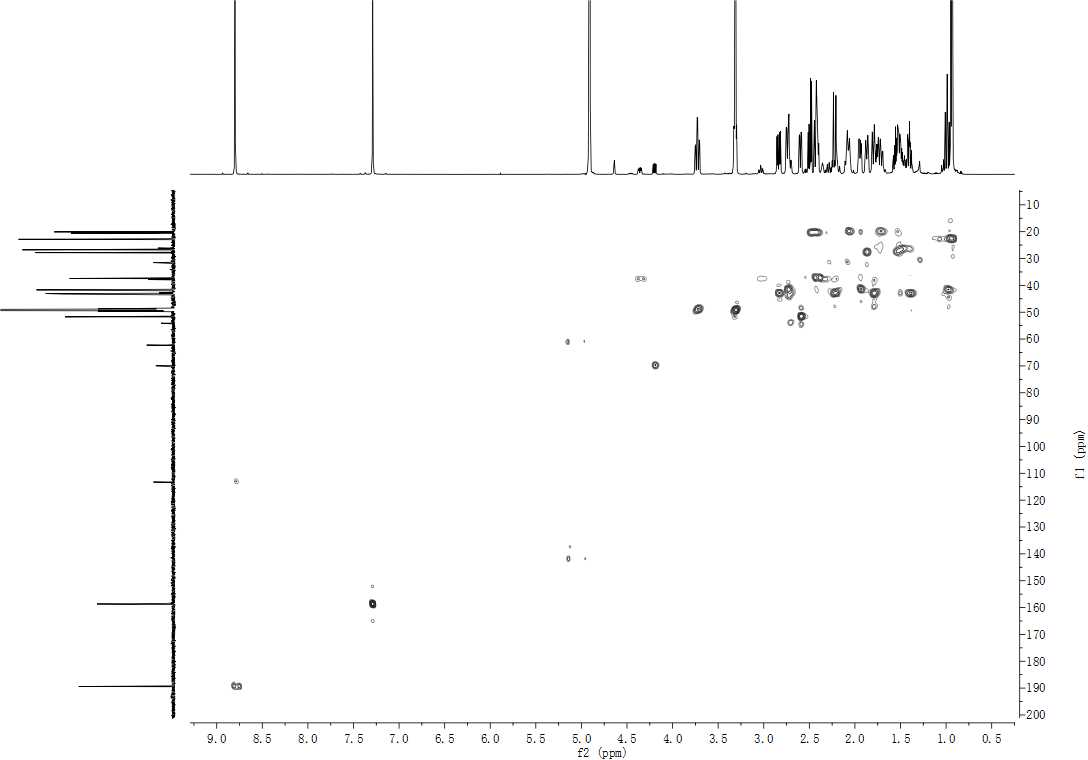


S5. ^1^H-^13^C HSQC NMR spectrum (methanol-*d_4_*) of compound **1**


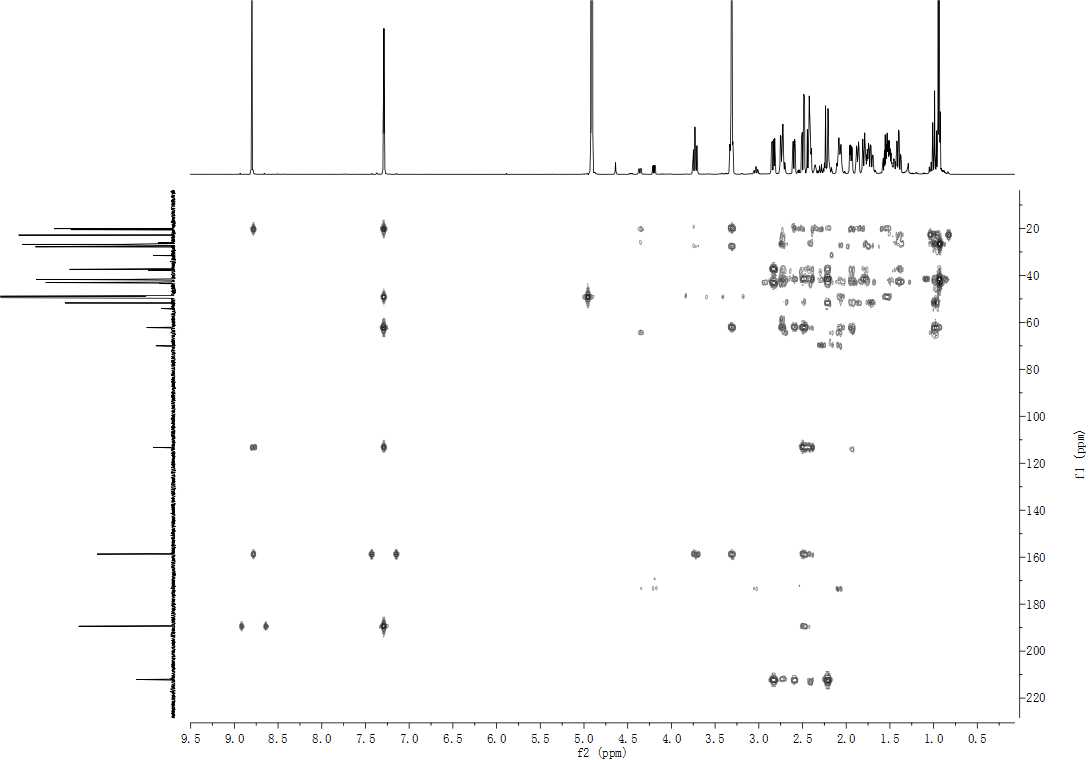


S6. ^1^H-^13^C HMBC NMR spectrum (methanol-*d_4_*) of compound **1**


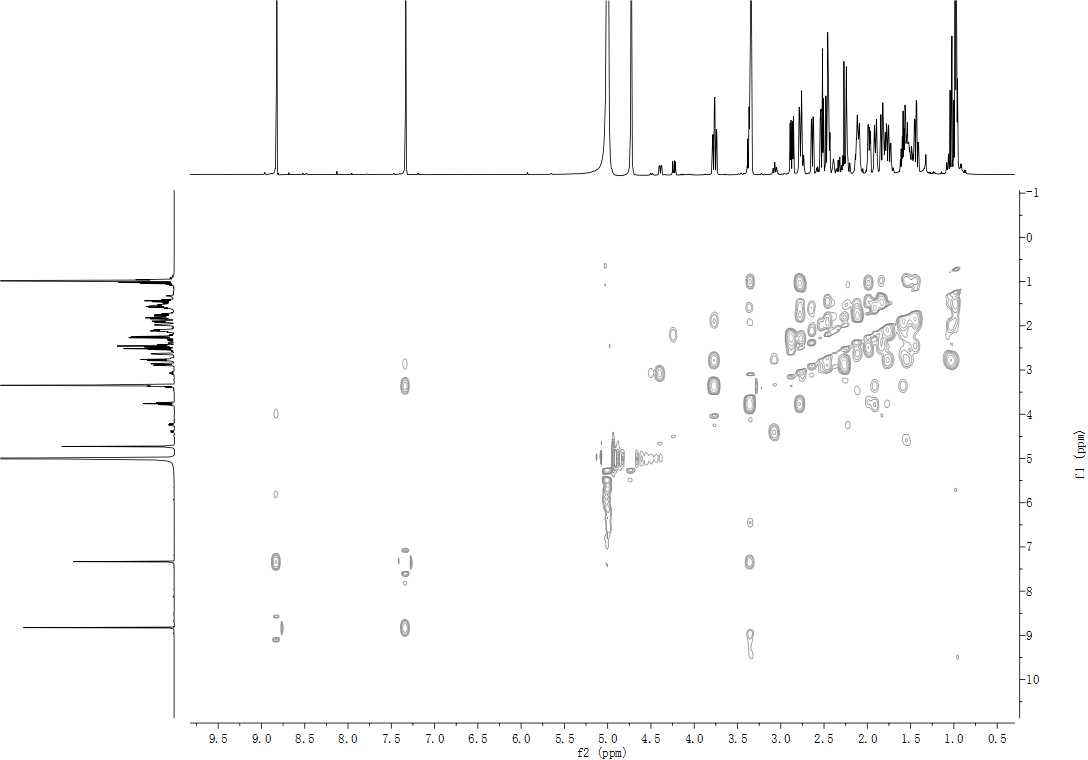


S7. ROESY spectrum (methanol-*d_4_*) of compound **1**


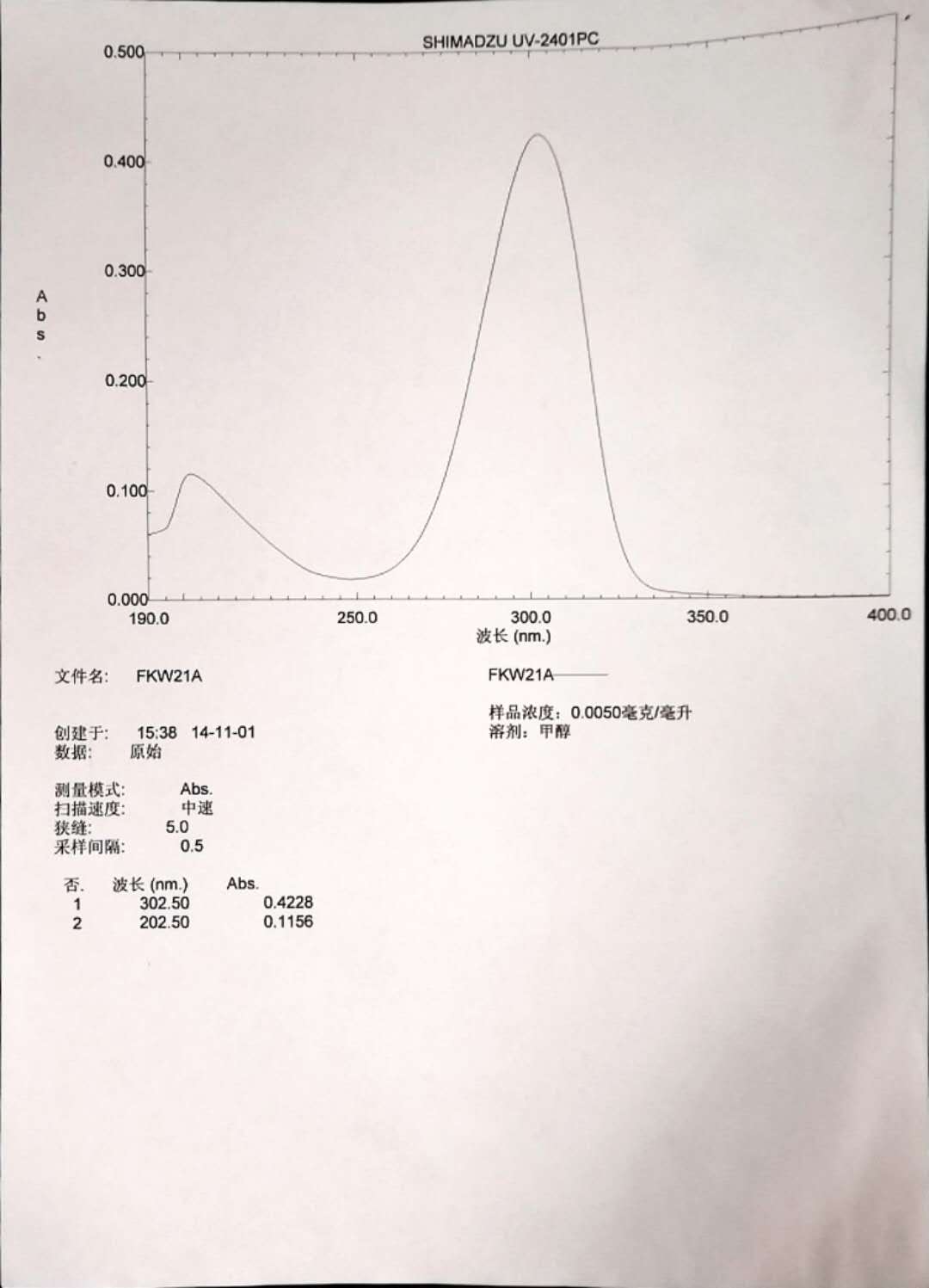


S8. UV spectrum of compound **1**


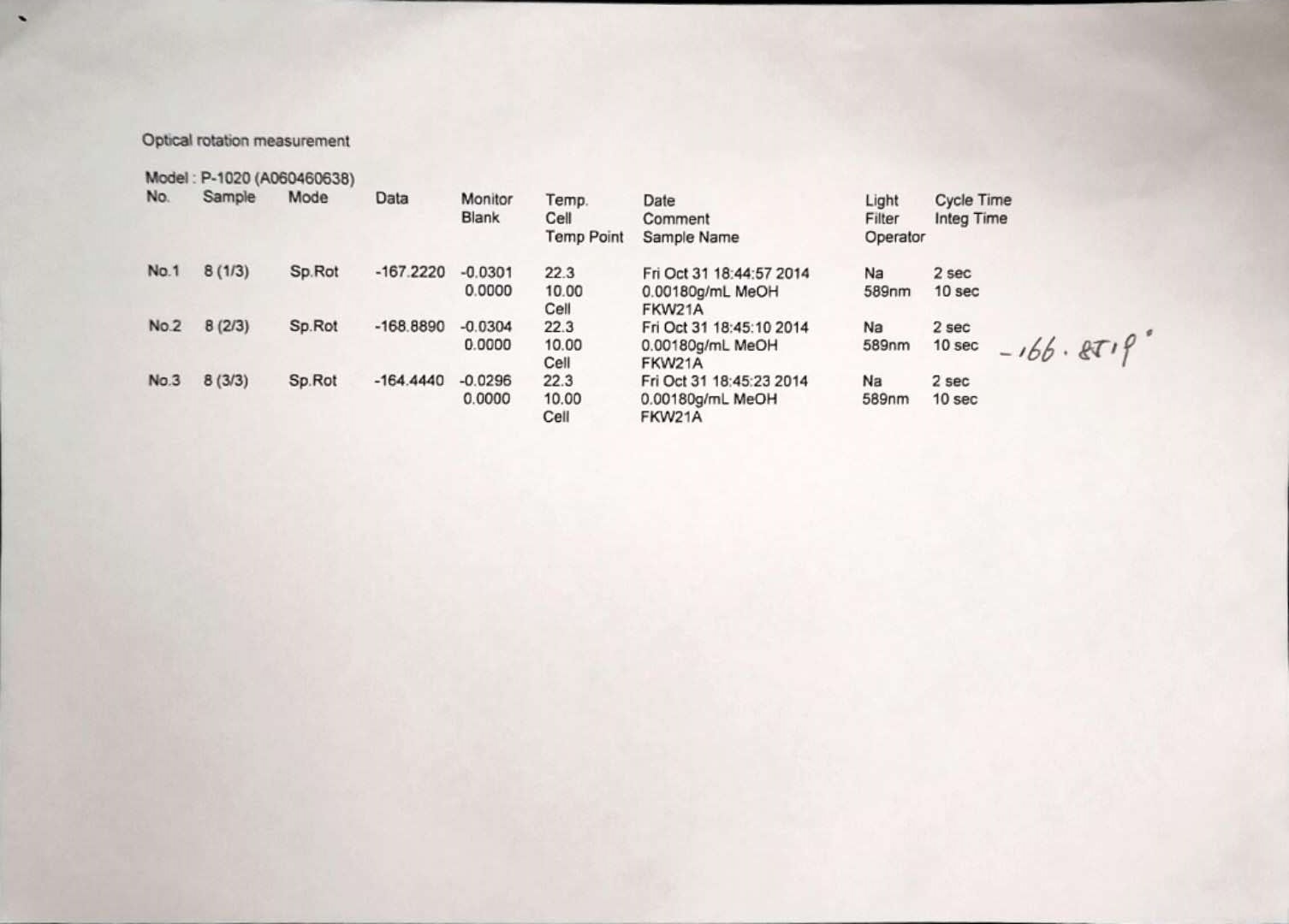


S9. OR spectrum of compound **1**


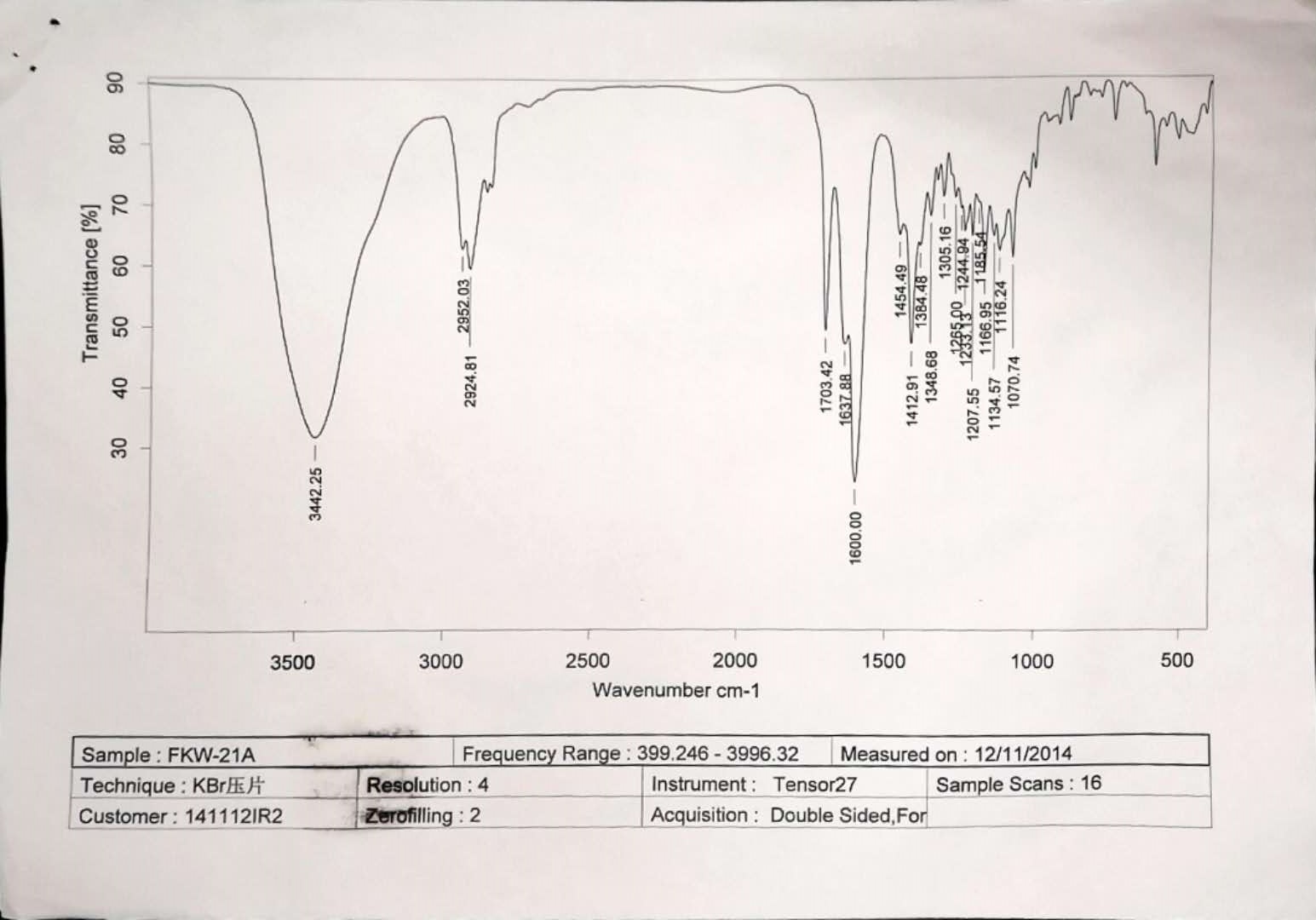


S10. IR spectrum of compound **1**


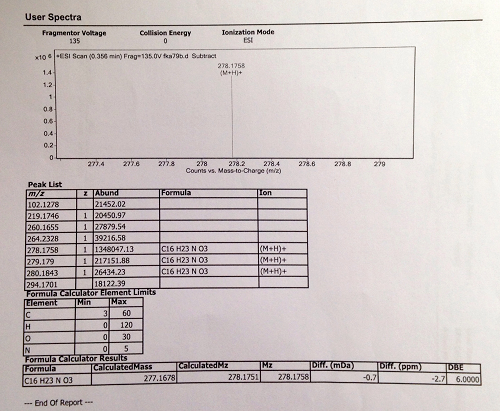


S11. HRESIMS spectrum of compound **2**

S12. ^1^H-NMR spectrum (600 MHz, CD_3_Cl) of compound **2**


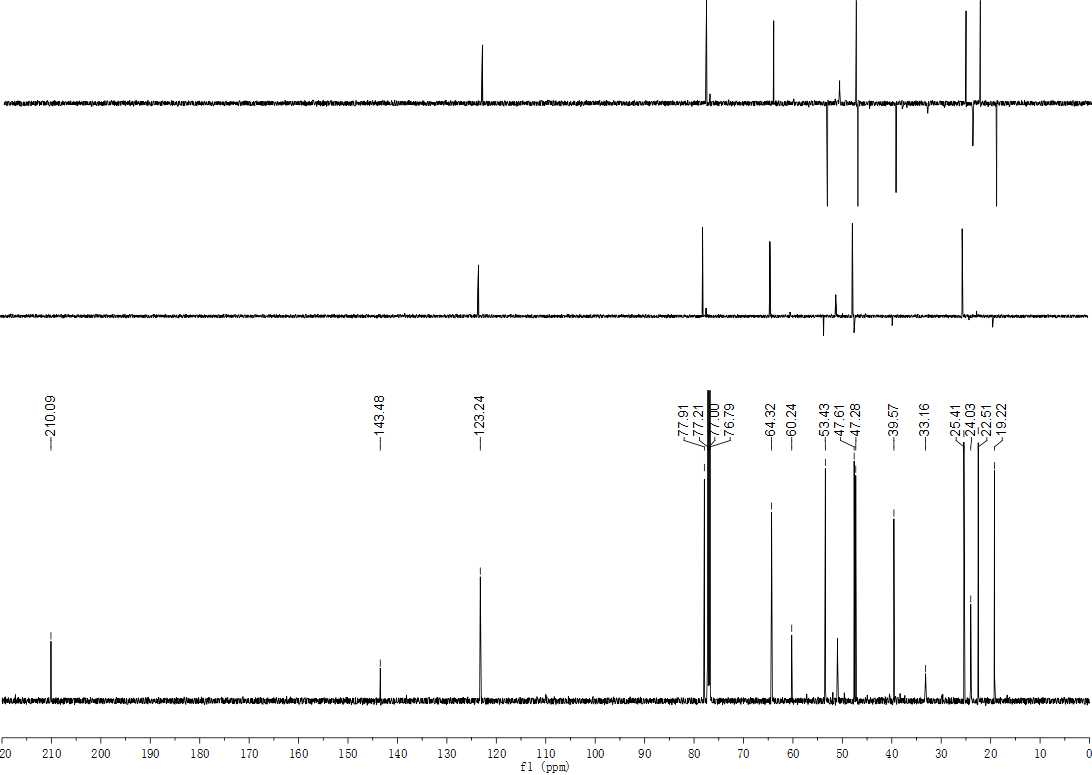


S13. ^13^C-NMR spectrum (150 MHz, CD_3_Cl) of compound **2**


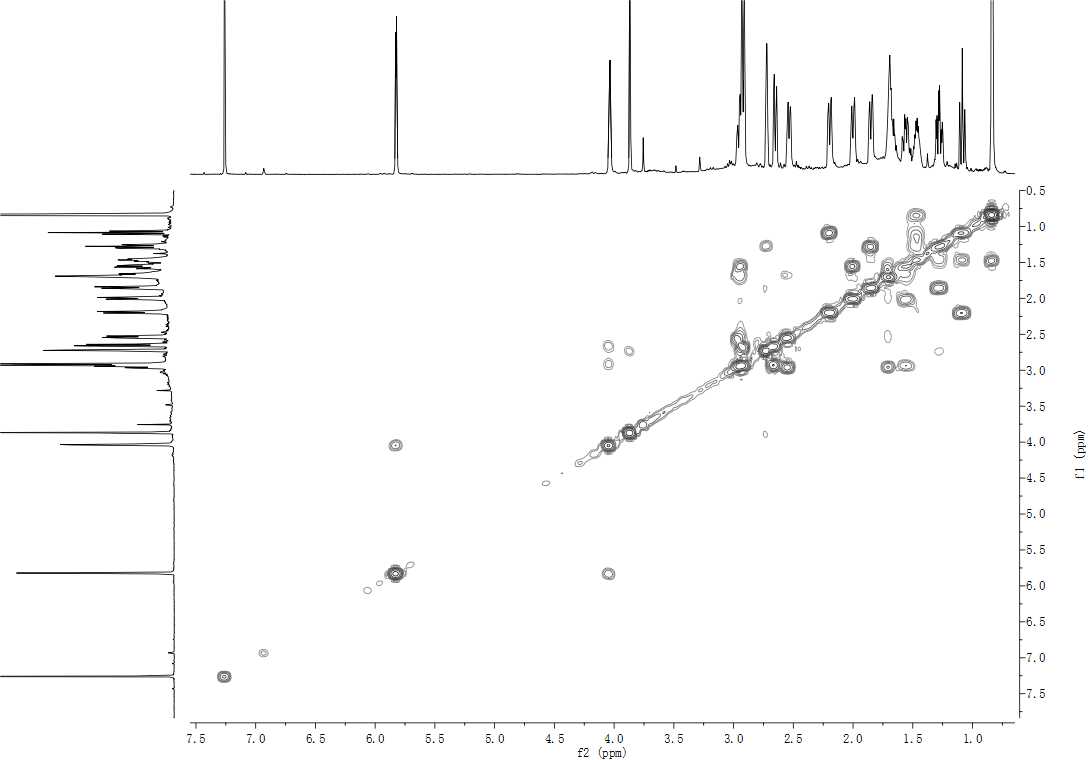


S14. ^1^H-^1^H COSY NMR spectrum (CD_3_Cl) of compound **2**


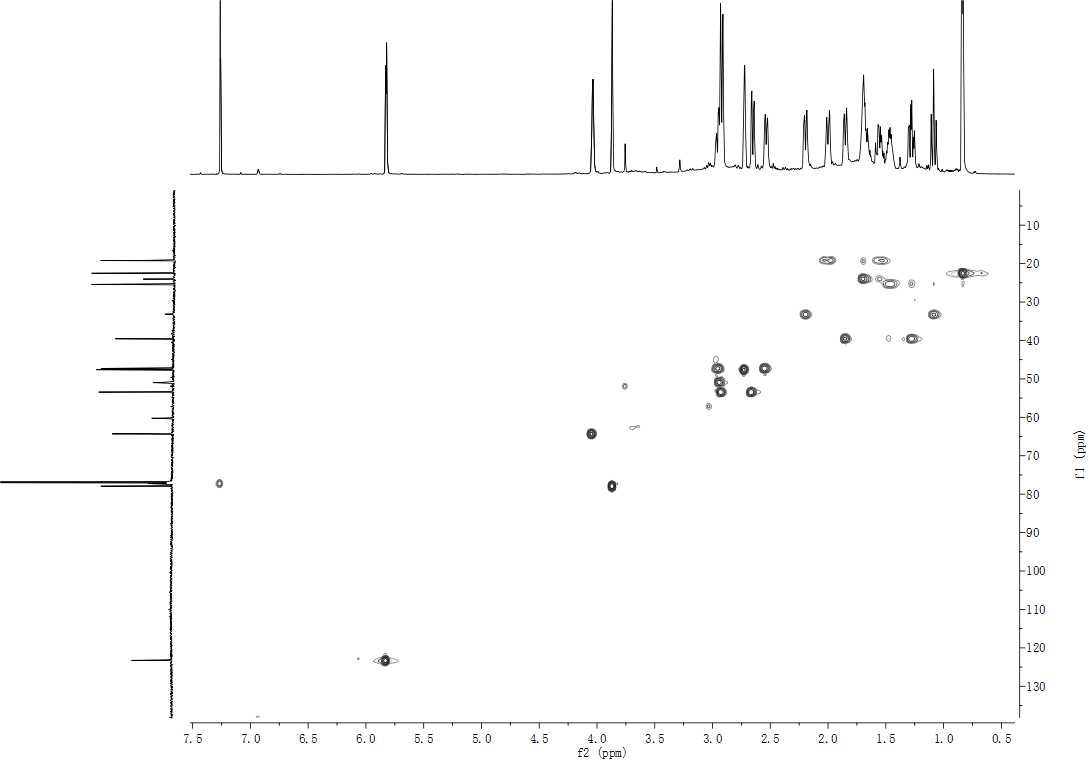


S15. ^1^H-^13^C HSQC NMR spectrum (CD_3_Cl) of compound **2**


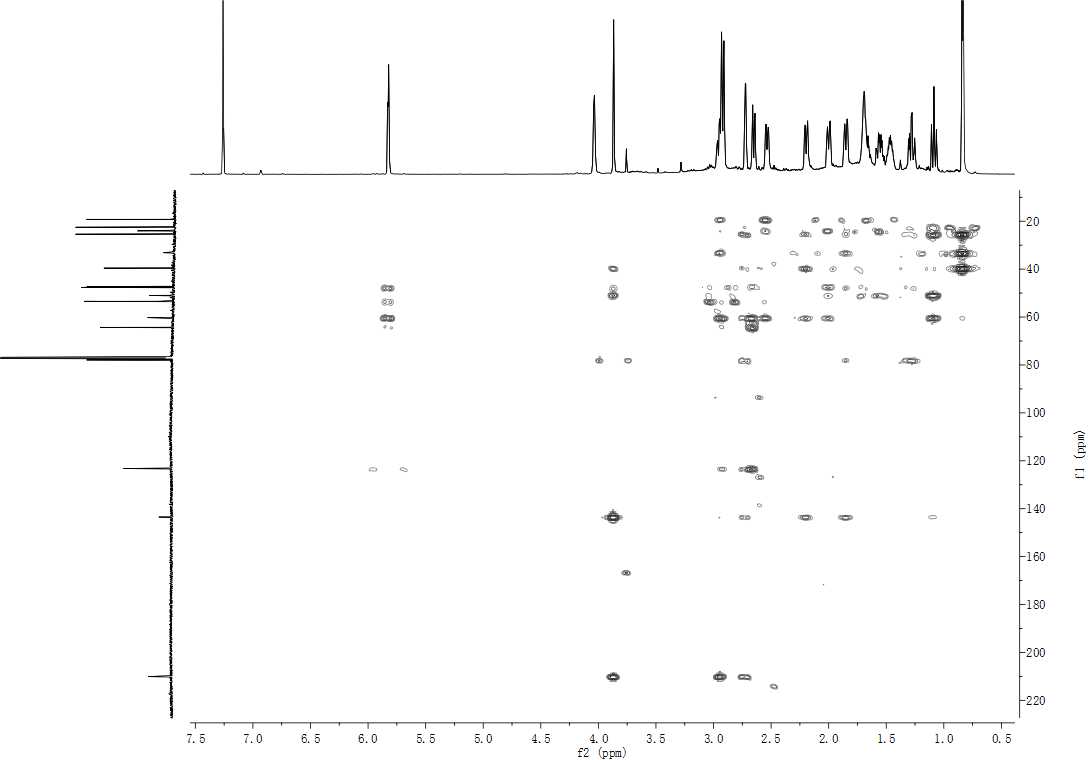


S16. ^1^H-^13^C HMBC NMR spectrum (CD_3_Cl) of compound **2**


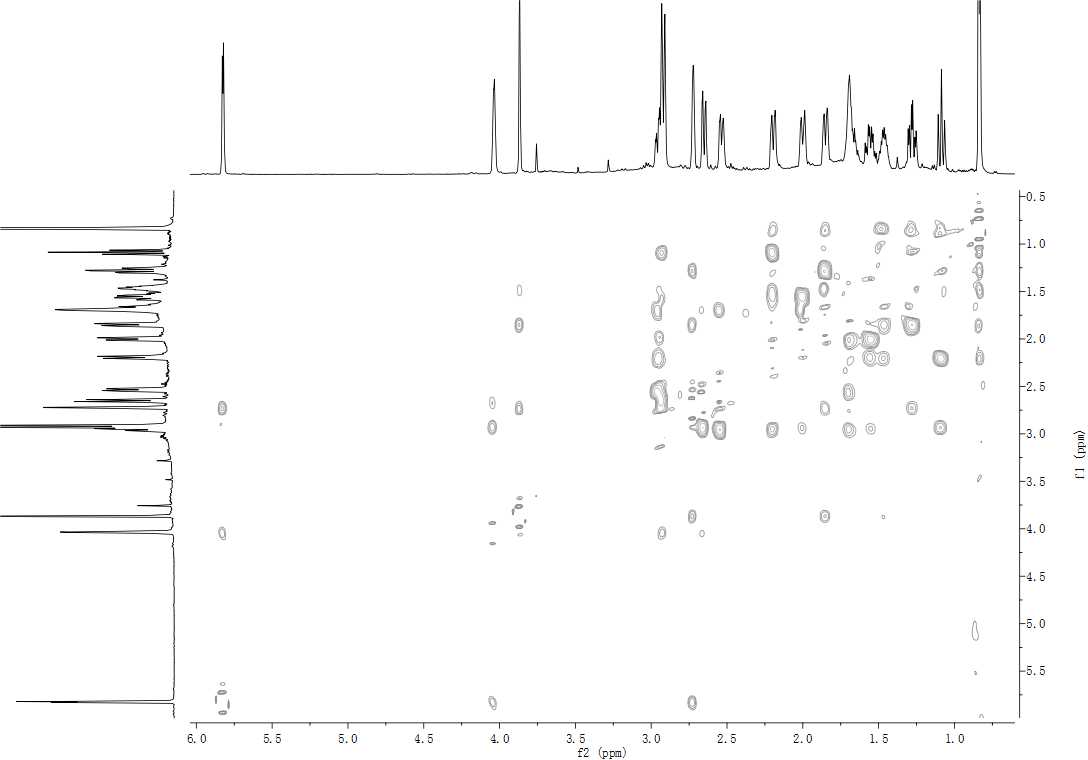


S17. ROESY spectrum (CD_3_Cl) of compound **2**


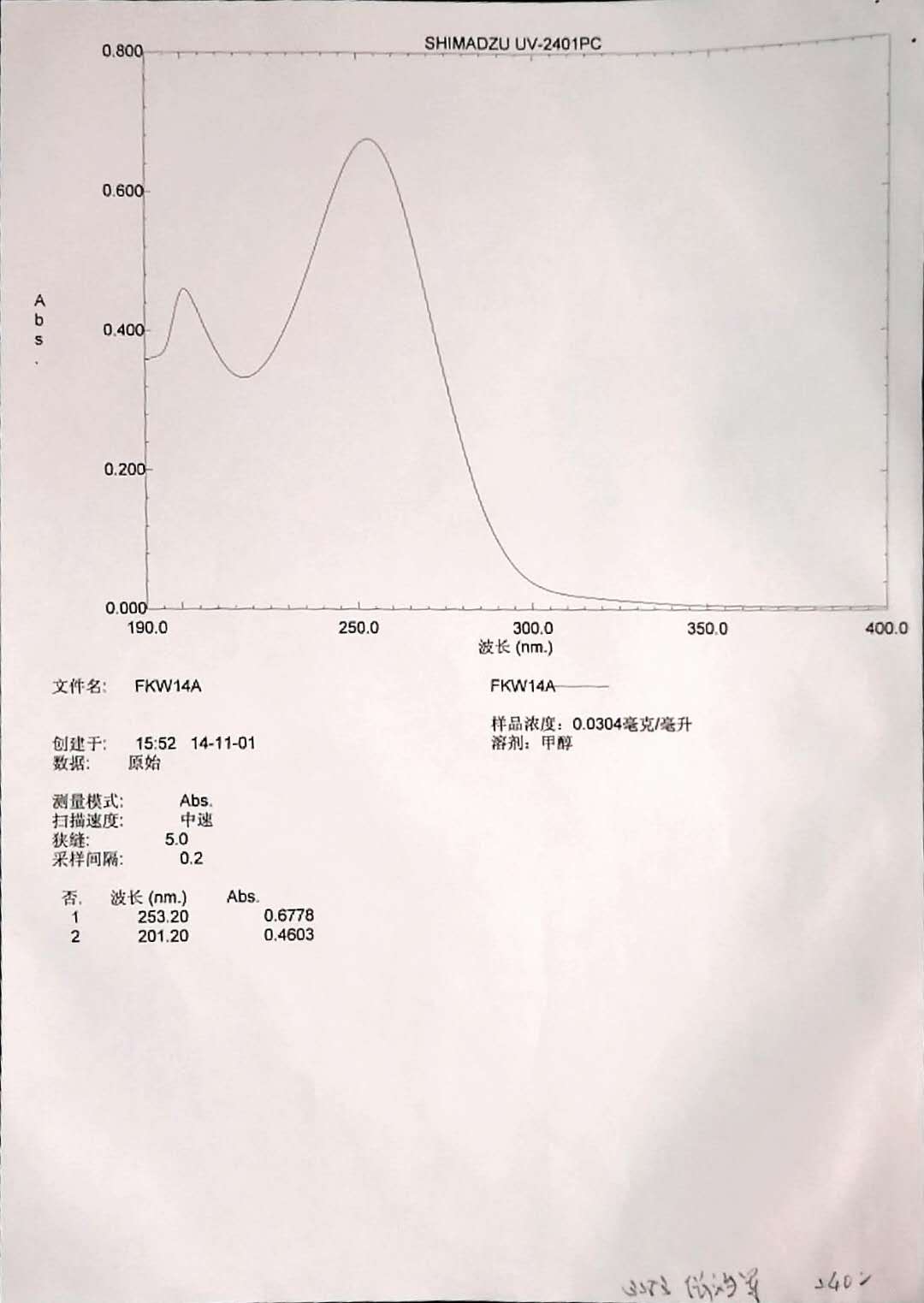


S18. UV spectrum of compound **2**


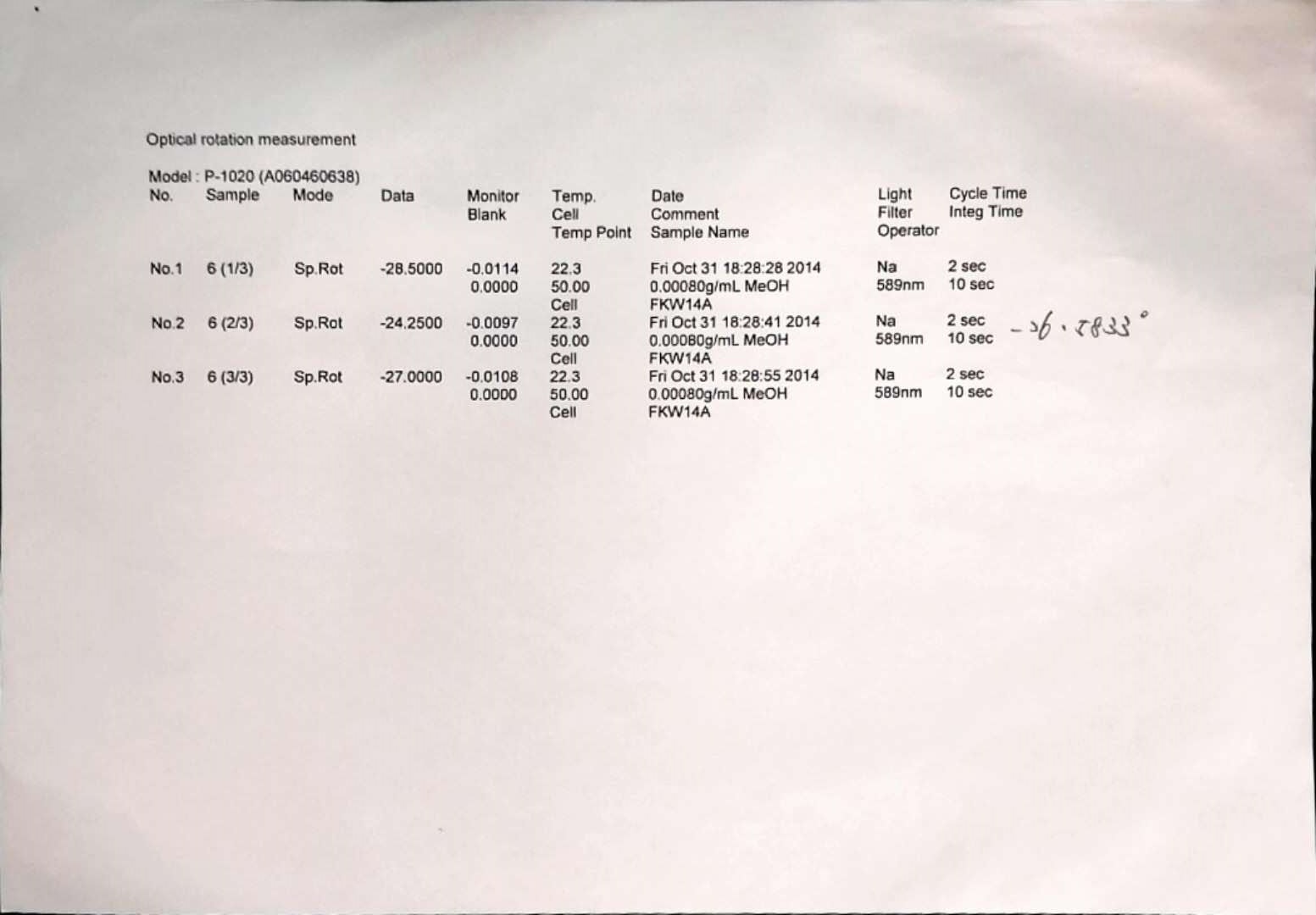


S19. OR spectrum of compound **2**


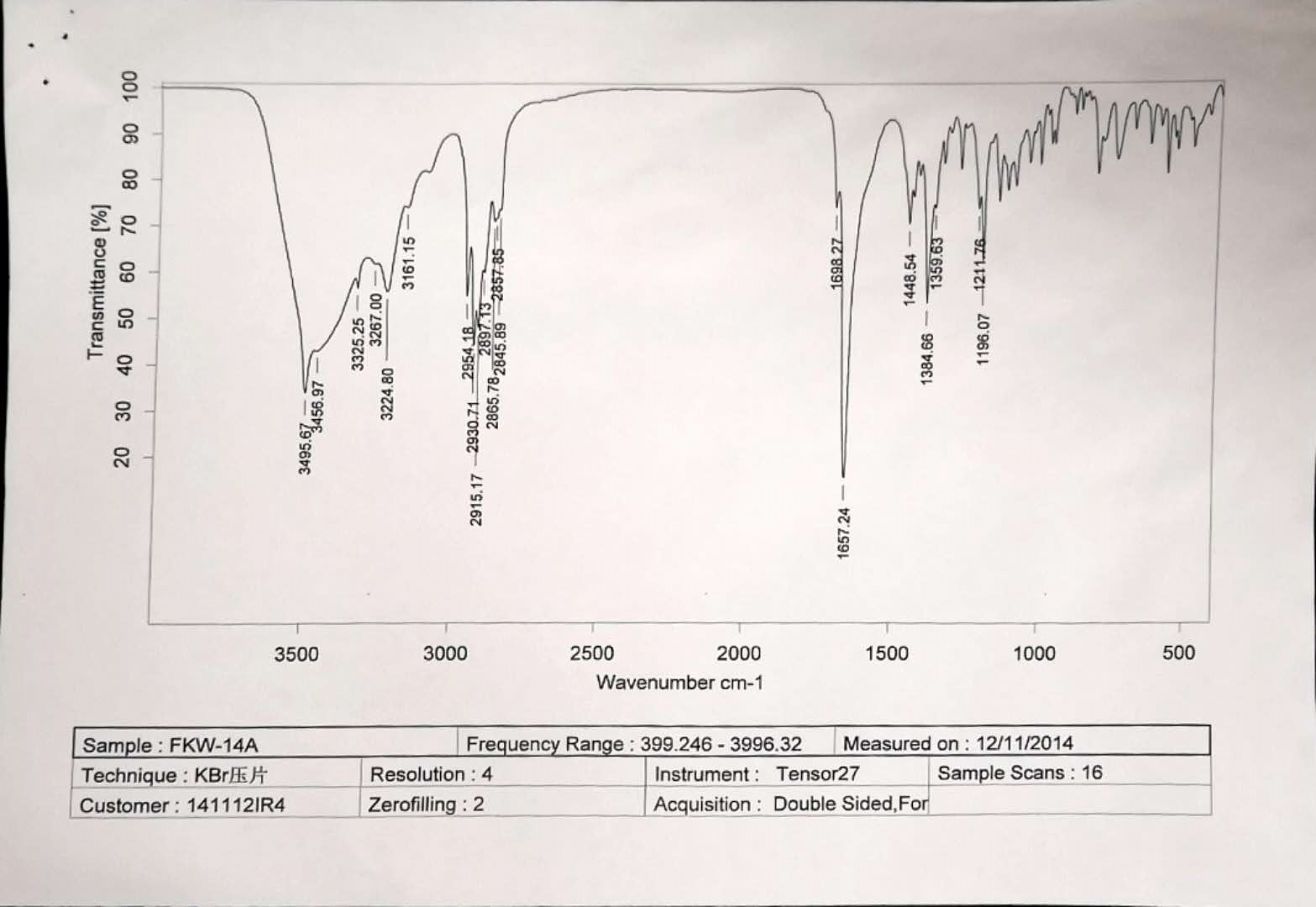


S20. IR spectrum of compound **2**


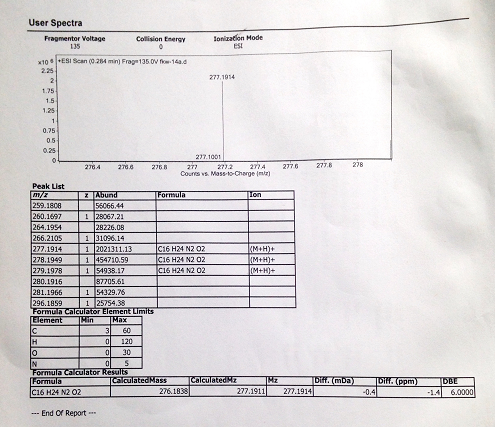


S21. HRESIMS spectrum of compound **3**


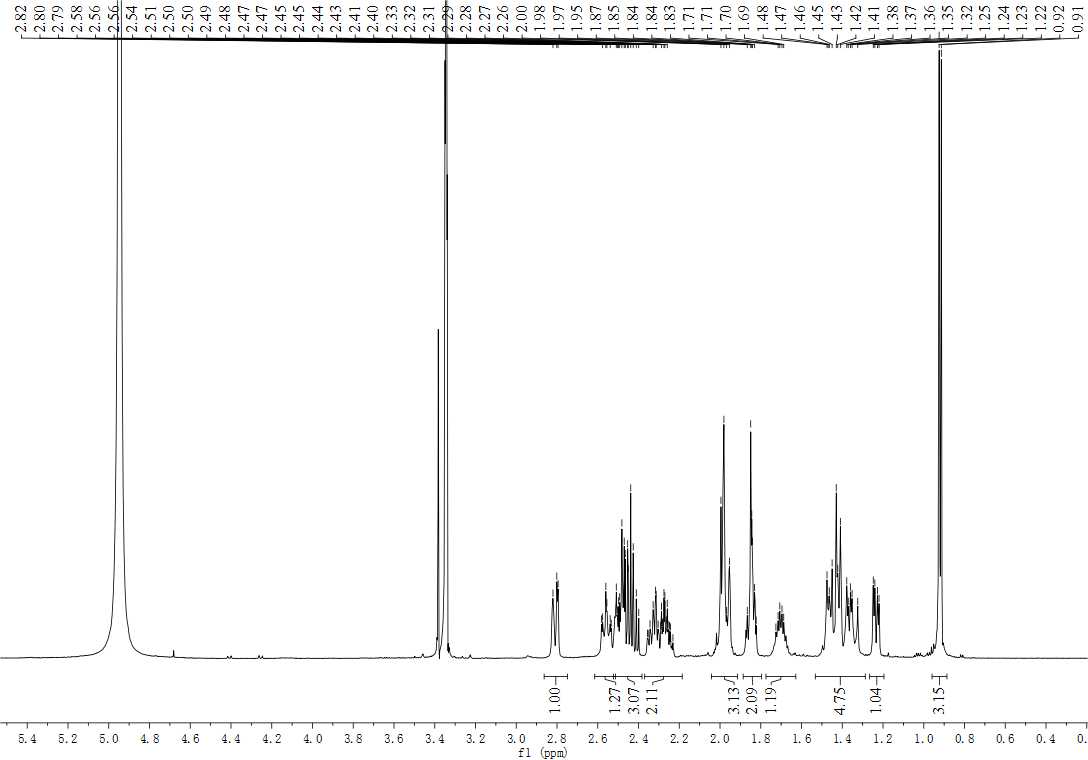


S22. ^1^H-NMR spectrum (600 MHz, methanol-*d_4_*) of compound **3**


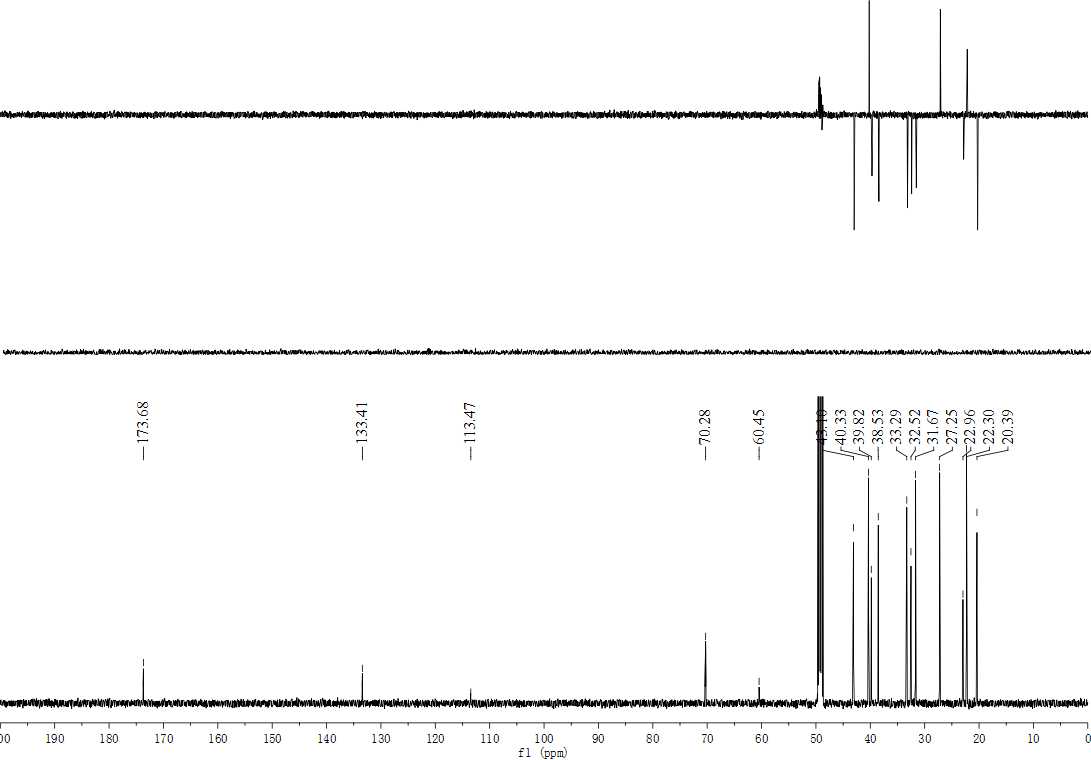


S23. ^13^C-NMR spectrum (150 MHz, methanol-*d_4_*) of compound **3**


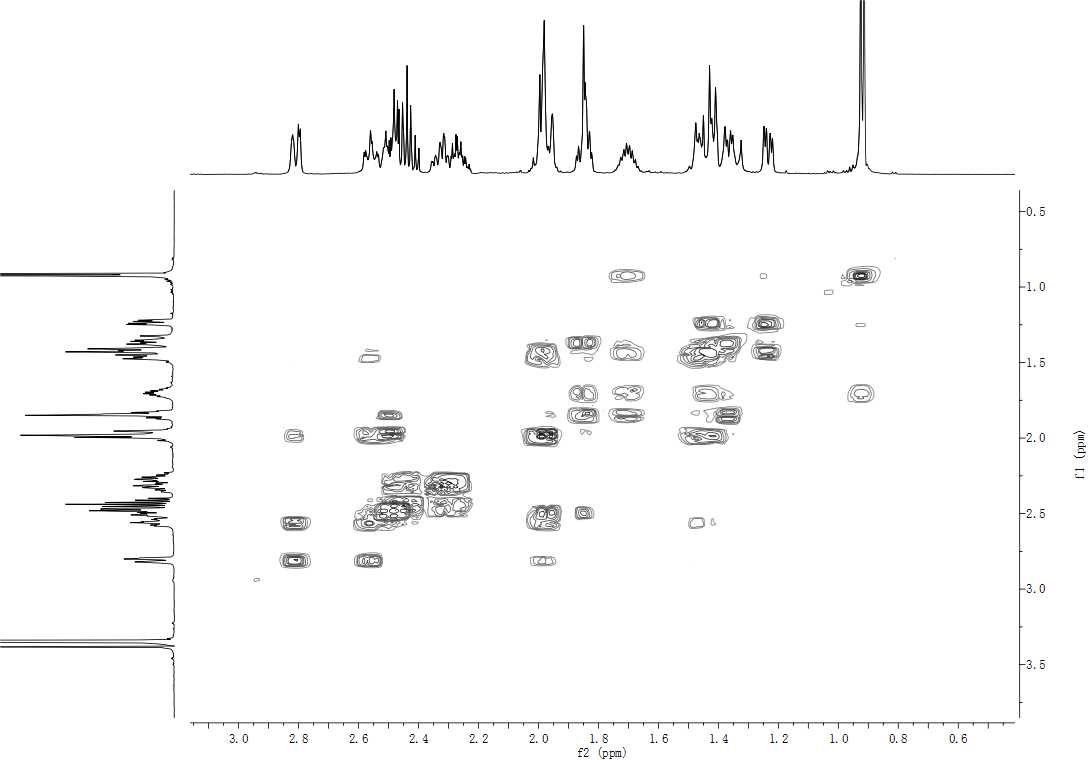


S24. ^1^H-^1^H COSY NMR spectrum (methanol-*d_4_*) of compound **3**


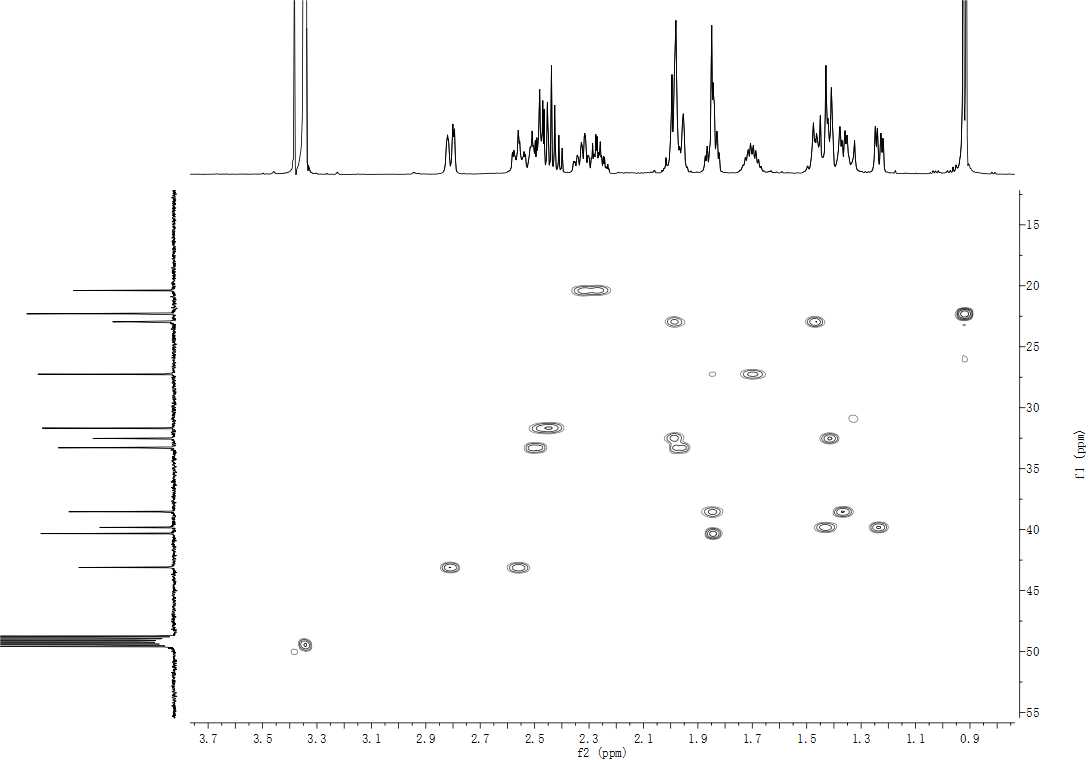


S25. ^1^H-^13^C HSQC NMR spectrum (methanol-*d_4_*) of compound **3**


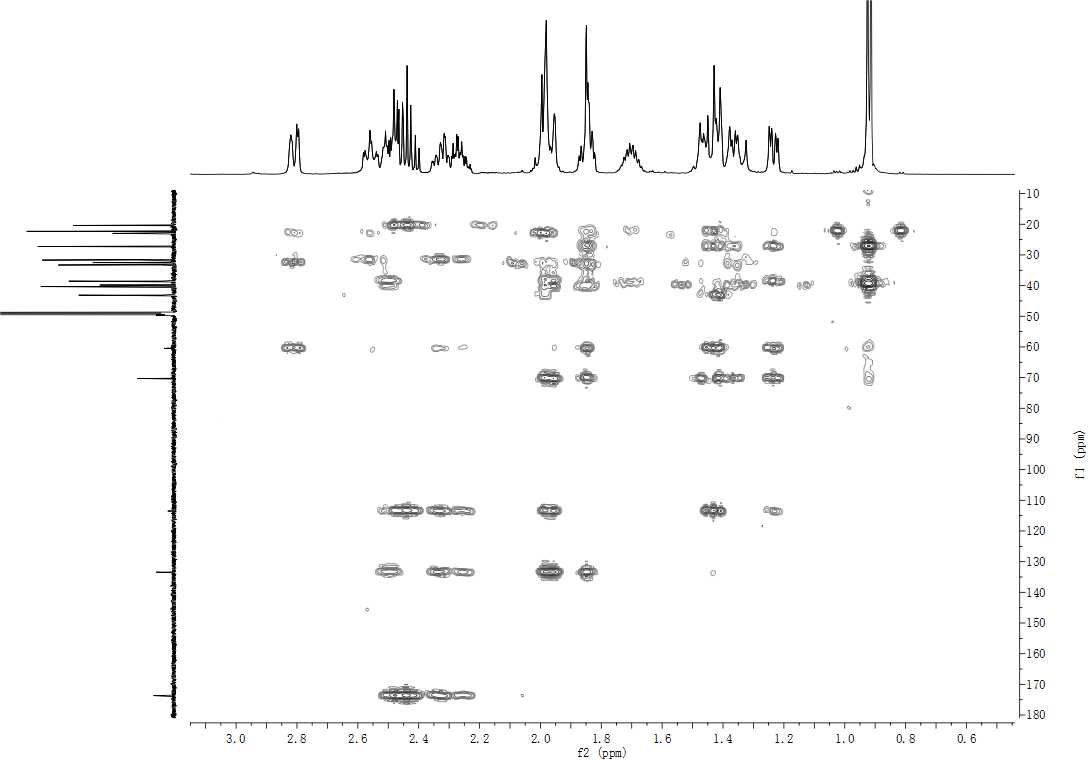


S26. ^1^H-^13^C HMBC NMR spectrum (methanol-*d_4_*) of compound **3**


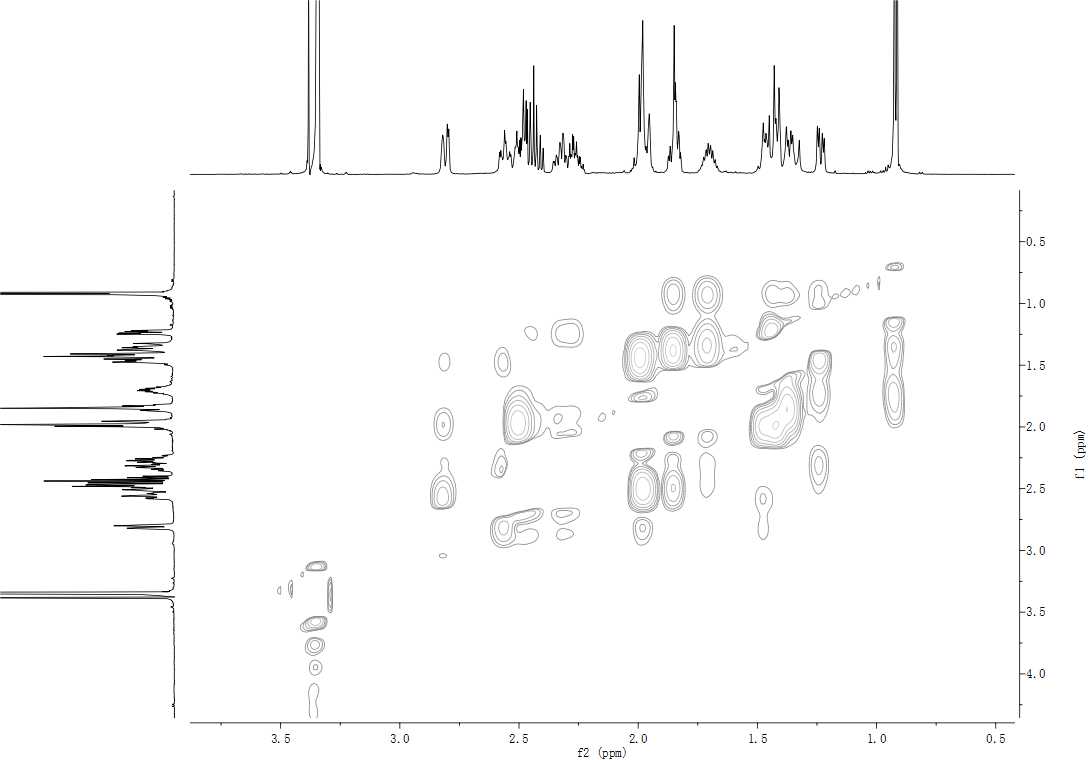


S27. ROESY spectrum (methanol-*d_4_*) of compound **3**


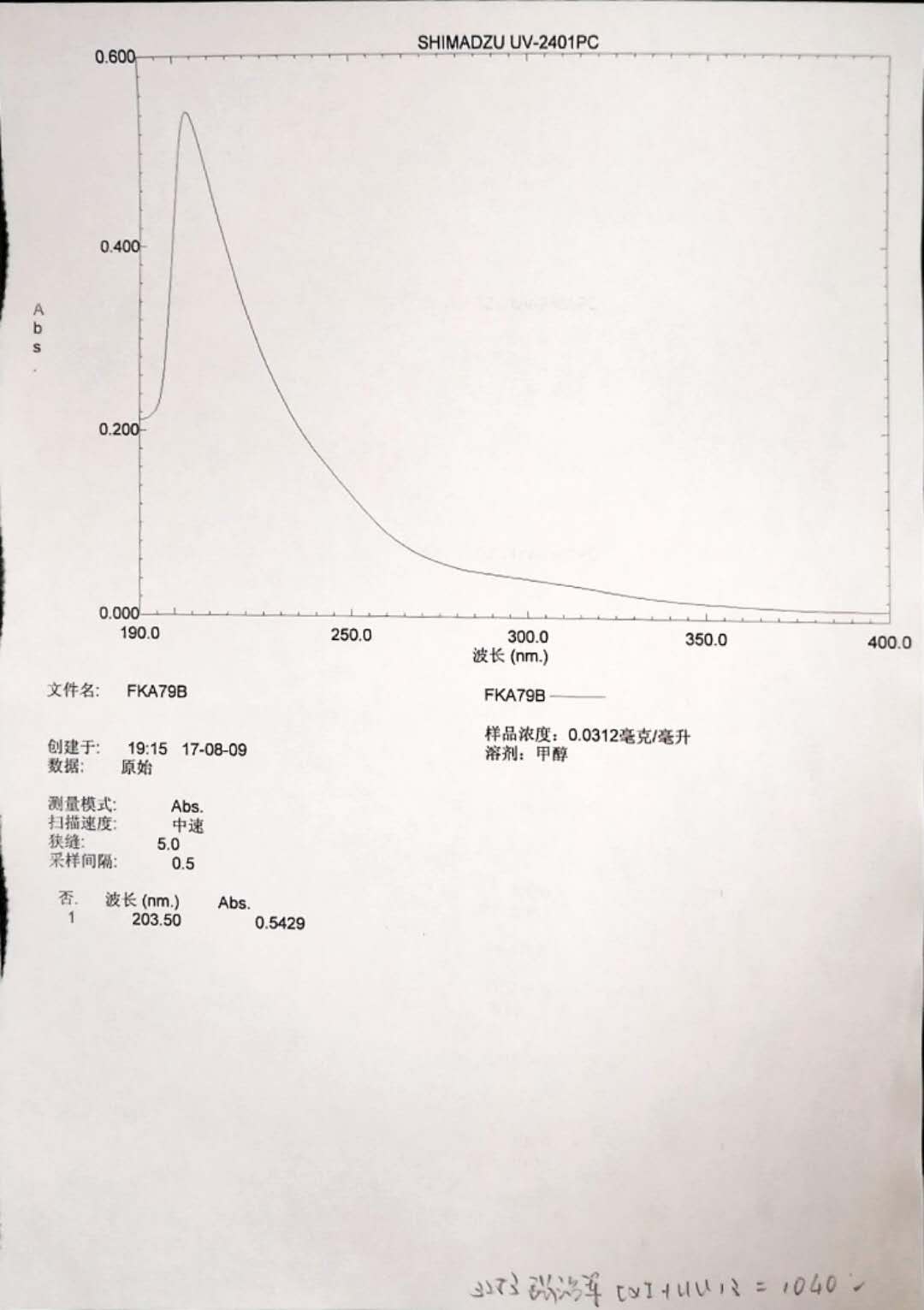


S28. UV spectrum of compound **3**


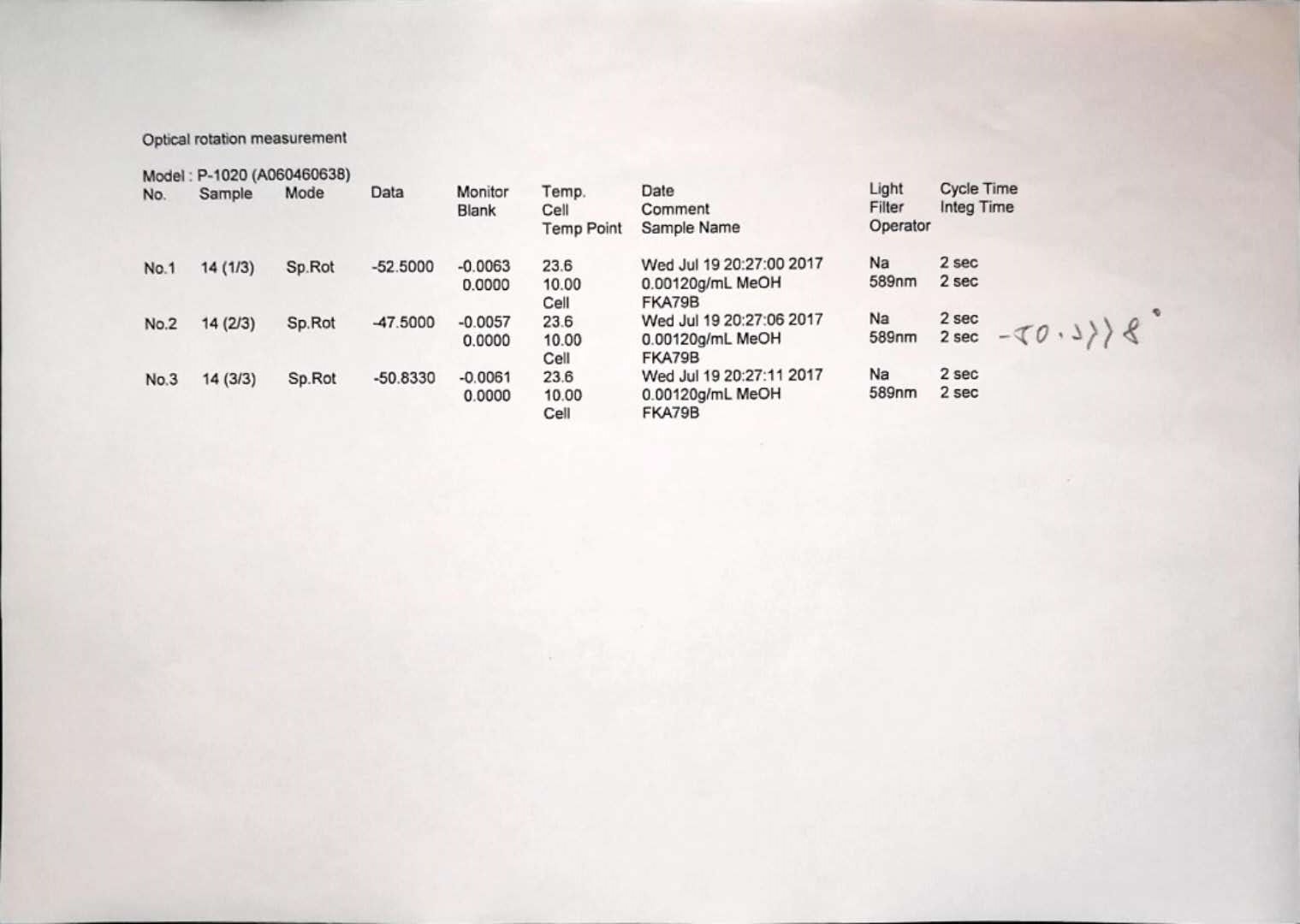


S29. OR spectrum of compound **3**


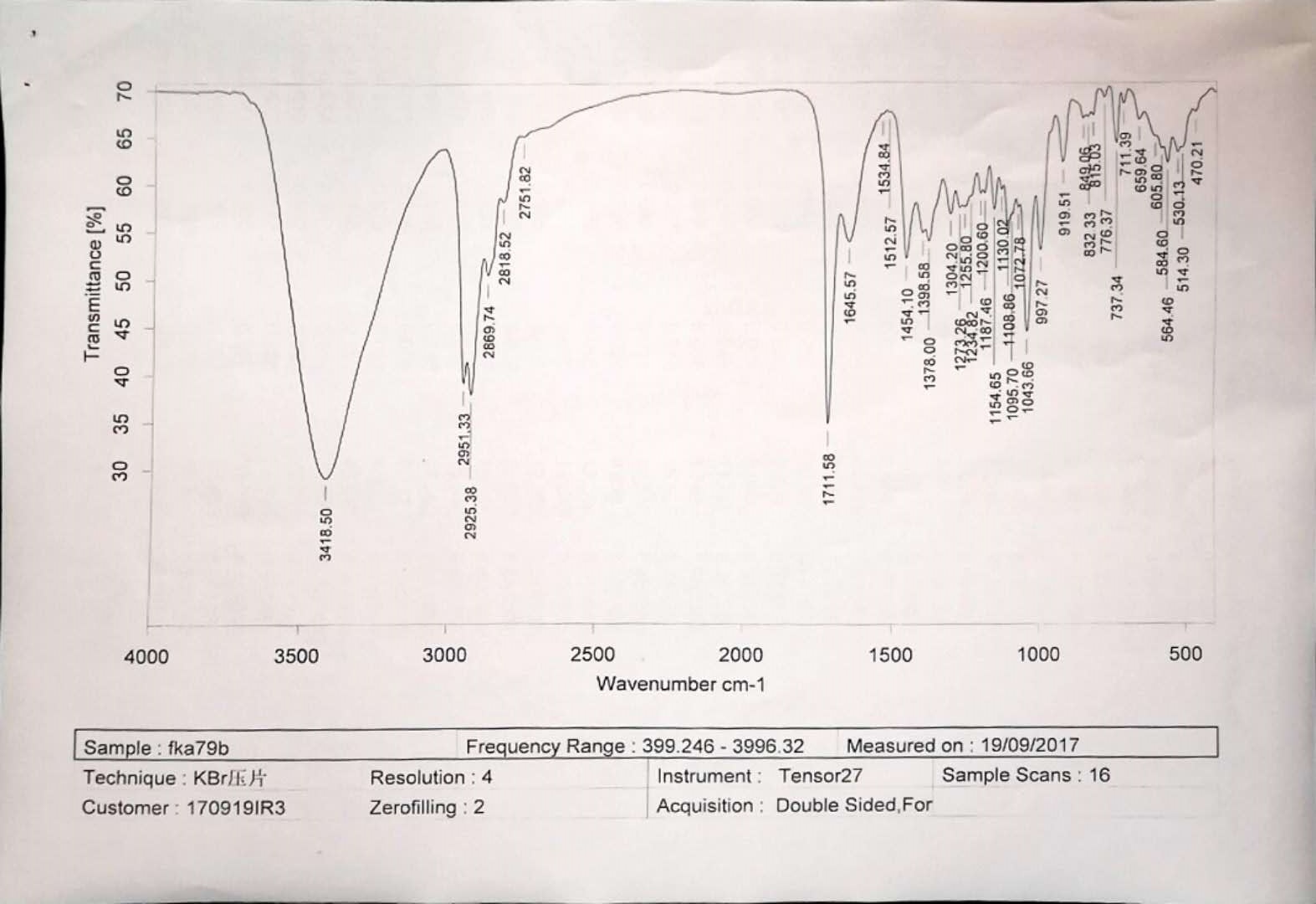


S30. IR spectrum of compound **3**

1.  Corresponding author. Tel.: +86-871-65223058; fax: +86-871-65215783;

   E-mail: [qinshizhao@mail.kib.ac.cn](mailto:qinshizhao@mail.kib.ac.cn) (Q.-S.Zhao) [↑](#footnote-ref-2)
